# Single-Crystal NMR for ^17^O in Alanine Enantiomers

**DOI:** 10.1021/acsphyschemau.5c00061

**Published:** 2025-11-12

**Authors:** Shiva Agarwal, Sungsool Wi, Jason Kitchen, Zhongrui Li, Christopher J. Taylor, Michael A. Famiano, John B. Miller

**Affiliations:** † Department of Physics, 4175 Western Michigan University, Kalamazoo, Michigan 49008, United States; ‡ 189689 National High Magnetic Field Laboratory, Tallahassee, Florida 32310, United States; ¶ Electron Microbeam Analysis Laboratory, 1259 University of Michigan, Ann Arbor, Michigan 48109, United States; § Department of Chemistry, 4175 Western Michigan University, Kalamazoo, Michigan 49008, United States

**Keywords:** amino acids, chirality, single-crystal NMR, shielding tensors, ^17^O, DFT

## Abstract

Single-crystal solid-state nuclear magnetic resonance (ssNMR) spectroscopy, which enables detailed analysis of the electronic structures of crystalline molecules, offers a unique opportunity to investigate molecular chirality—an essential feature with broad implications for understanding the origin and function of life. In this study, we employ single-crystal ssNMR spectroscopy, in combination with X-ray diffraction and density functional theory (DFT) calculations, to examine the electronic structure of ^17^O nuclei in crystalline forms of alanine enantiomers. Eight magnetically nonequivalent ^17^O resonances within the unit cell were observed and successfully assigned, and their corresponding NMR tensor parameters were determined. These resonances are comprised of pairs of chemically distinct oxygens in each of four symmetrically related sites. The experimental findings were compared with previous NMR studies as well as with DFT calculations performed in this work. The DFT results not only supported the assignment of crystallographically distinct ^17^O sites but also revealed previously unobserved antisymmetric components of the chemical shift tensors. This study presents the first comprehensive characterization of ^17^O NMR tensors in alanine enantiomers and underscores the power of integrating single-crystal ssNMR with X-ray diffraction and DFT calculations to advance our understanding of molecular chirality in amino acids.

## Graphical abstract

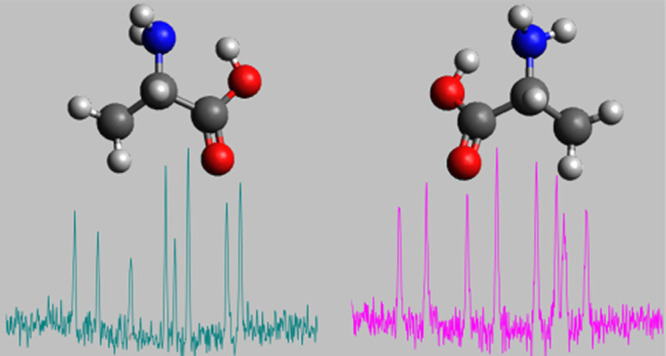

## Introduction

Biological homochirality is a fundamental feature of terrestrial life.^1,2^ Analysis of a number of carbonaceous meteorites have revealed an excess of l-amino acids compared to the d *-*enantiomers.^3–5^ These findings suggest that abiotic mechanism(s) operating in the stellar environments may be responsible for generating this enantiomeric excess (*ee*).^6^ Several abiotic models^7–11^ have been proposed to explain the observed *ee* of amino acids. Among them, the magnetochiral model calculates *ee* of as high as 0.02% for alanine, positive *ee* for many α-amino acids, and up to 0.01% for cationic isovaline and zwitterionic alanine.^11–13^

The orientation dependence arising from optical isomerism influences the relative nuclear interaction rates of chiral ^14^N nuclei in amino acids with relativistic leptons, such as electron antineutrinos (ν̅_e_), potentially leading to their conversion into ^14^C. This mechanism may contribute to the preferential destruction of d-amino acids over their l-counterparts. In single-crystal ssNMR experiments, the orientation of chiral molecules in high magnetic fields affects the electronic environment such that the antisymmetric components of the magnetic shielding tensor are altered to exhibit reflection symmetry.

The initial motivation of this work was to experimentally test the predictions of the magnetochiral model. The primary aim was to examine the antisymmetric chemical shielding (ACS) tensor components of the ^14^N nucleus in amino acid samples. That target has proven elusive (see Results and Discussion). Instead, we turned to the oxygen atoms in the carboxylate moiety of the amino acids. Of the three stable oxygen isotopes: ^16^O, ^17^O, and ^18^O, only ^17^O (*I* = 5/2) is NMR active. Being a quadrupolar nucleus as well, ^17^O can serve as a proxy for the ^14^N as a probe of the chiral environments of most amino acids.

While ^17^O NMR studies in solution have provided valuable insights,^14,15^ solution studies alone cannot fully capture the detailed electronic and structural information on these systems; too much information is lost due to randomized molecular orientations. ACS contributions to the CSA have been indirectly inferred through relaxation studies in solution-state NMR.^16,17^ Some aspects of electronic structure, such as quadrupolar coupling and chemical shield anisotropy (CSA), can be obtained through solid-state NMR.^18^ For quadrupolar nuclei (*I* > 1/2), the quadrupolar and ACS components are coupled to each other and this can also be measured using solid-state NMR spectroscopy.^19,20^ To achieve a more complete understanding of the molecular structure of amino acids, and to better characterize the electronic state of oxygen in biologically important molecules, advanced solid-state NMR techniques such as single-crystal NMR (SCNMR) must be utilized.^21^

In this work, we have investigated the quadrupolar tensor and the chemical shielding anisotropy tensor for ^17^O nuclei in both enantiomers of isotopically enriched alanine. The relative orientations of these tensors were determined through SCNMR spectroscopy. Due to its low natural abundance (∼0.04%), isotopically enriched samples are required for rapid and facile ^17^O NMR spectroscopy experiments.^22^ The experimental findings were compared with previously reported NMR studies on alanine enantiomers,^23–25^ as well as with periodic density functional theory (DFT) calculations using the published crystal structures.^26^ The DFT models were performed using the PBEsol exchange-correlation functionals.^27^ The NMR tensors were calculated using the gauge-including projector augmented wave (GIPAW) method.^28^ We also present similar calculated data for the ^14^N nuclei.

## Theory

The NMR rotating frame Hamiltonian of an isolated quadrupolar nucleus, considered in the Zeeman interaction frame includes contributions from chemical shielding and quadrupolar interactions and is expressed as^29^

$${\hat{H}}_{\mathrm{Rot}}^{\mathrm{Total}}={\hat{H}}_{Q}^{\left(1\right)}+{\hat{H}}_{Q}^{\left(2\right)}+{\hat{H}}_{\mathrm{CSA}}^{\left(1\right)}$$ 1

with

$$\begin{array}{ll} {\hat{H}}_{Q}^{\left(1\right)}=\chi_{Q}R_{2,0}^{Q}\{3I_{z}^{2}-I(I+1)\} & \;\left(∵\chi_{Q}=\frac{eQ}{2I(2I-1)\hbar }\right) \end{array}$$ 2

$${\hat{H}}_{Q}^{\left(2\right)}=\frac{1}{2\omega_{0}}\chi_{Q}^{2}[R_{2,-1}^{Q}R_{2,1}^{Q}I_{z}\{4I(I+1)-8I_{z}^{2}-1\}+R_{2,-2}^{Q}R_{2,2}^{Q}I_{z}\{2I(I+1)-2I_{z}^{2}-1\}]$$ 3

and

$${\hat{H}}_{\mathrm{CSA}}^{\left(1\right)}=\{\delta_{\mathrm{iso}}+R_{2,0}^{\mathrm{CSA}}\}\gamma B_{o}I_{z}$$ 4

In the above equations, ${\hat{H}}_{Q}^{\left(1\right)}$ , ${\hat{H}}_{Q}^{\left(2\right)}$ , ${\hat{H}}_{\mathrm{CSA}}^{\left(1\right)}$ represent the first order quadrupolar interaction, second-order quadrupolar interaction, and first order chemical shift anisotropy, respectively. The term δ_iso_ denotes the isotropic chemical shift, and the components *R*_2,λ_^ξ^ (with ξ = CSA or Q, and λ = 2, 1, 0, −1, or −2) represent the spatial part of tensors defined in the laboratory (rotating) frame. Here, *I* is the nuclear spin quantum number, *I*_*z*_ is the *z*-component of the angular momentum operator, and *eQ* is the nuclear quadrupole moment, γ is the gyromagnetic ratio, and ω_0_ is the nuclear Larmor frequency. The spherical tensors *R*_2,λ_^ξ^, defined in the laboratory frame, can be related to the corresponding *G*_2,λ_^ξ^ tensors defined in the goniometer-tenon frame through a single-step coordinate transformation using the polar angle θ and an azimuthal angle ϕ according to^19,30^

$$\begin{array}{ll} \mathrm{Goniometer}\;\mathrm{frame}\overset{\left(\phi ,\;\theta ,\;0{}^{\circ}\right)}{\to }\mathrm{Laboratory}\;\mathrm{frame} & \left(B_{o}\;\mathrm{along}\;\mathrm{the}\;z‐\mathrm{axis}\right) \end{array}$$ 5

After explicitly performing this transformation and re-expressing the spherical tensors *G*_2,λ_^ξ^ into Cartesian tensors *G*_mn_^ξ^ (where m and n are *x*, *y*, or *z*) for easier interpretation, the expression for *R*_2,λ_^ξ^ and *R*_2,λ_^ξ^ *R*_2λ*′*_^ξ^ can be written as functions of *G*_mn_^ξ^, θ, and ϕ, as described previously.^31^

As shown in Figure 1 below, the goniometer tenon, to which the sample crystal is glued for mounting in the SCNMR probe, is designed to allow rotations about the −*x*, *y*, and −*z* axes. The relevant expressions used to interpret the −*x* ^*T*^ rotation (θ = −Θ; ϕ = π/2), *y* ^*T*^ rotation (θ = Θ; ϕ = 0), and −*z* ^*T*^ rotation (θ = π/2; ϕ = −Θ) patterns for the transitions of ^17^O (*I* = 5/2) can be derived from the eqs 2–4 above by considering transitions among different energy levels. Here, Θ represents the rotation angle applied experimentally by rotating the crystal about an axis that is oriented 90° relative to the external magnetic field (see Figure 1). For the central transition |1/2⟩↔|−1/2⟩, all three rotation patterns can be rearranged into the following expressions for explicit curve fitting, incorporating the first-order CSA and second-order quadrupolar contributions:

$$\nu_{\left|1/2\rangle ↔|-1/2\right\rangle }^{\mathrm{CSA}}=A^{\mathrm{CSA}}+B^{\mathrm{CSA}}\cos\;2\Theta +C^{\mathrm{CSA}}\sin\;2\Theta$$ 6

$$\nu_{\left|1/2\rangle ↔|-1/2\right\rangle }^{Q2}=A^{Q}+B^{Q}\cos\;2\Theta +C^{Q}\sin\;2\Theta +D^{Q}\cos\;4\Theta +E^{Q}\sin\;4\Theta$$ 7

where α ∈ {−*x* ^*T*^, *y* ^*T*^, −*z* ^*T*^} and the coefficients Γ_*m*_^ξ^ (with Γ = *A*, *B*, *C*, ···, *E*) are defined in terms of the *G*_mn_^ξ^ tensors,^31^ which are (3 × 3 matrices). Note that the dominant first-order quadrupolar Hamiltonian vanishes for symmetric transitions such as |1/2⟩↔|−1/2⟩, due to its quadratic dependence on *I*_*z*_^2^.

**Figure 1:**
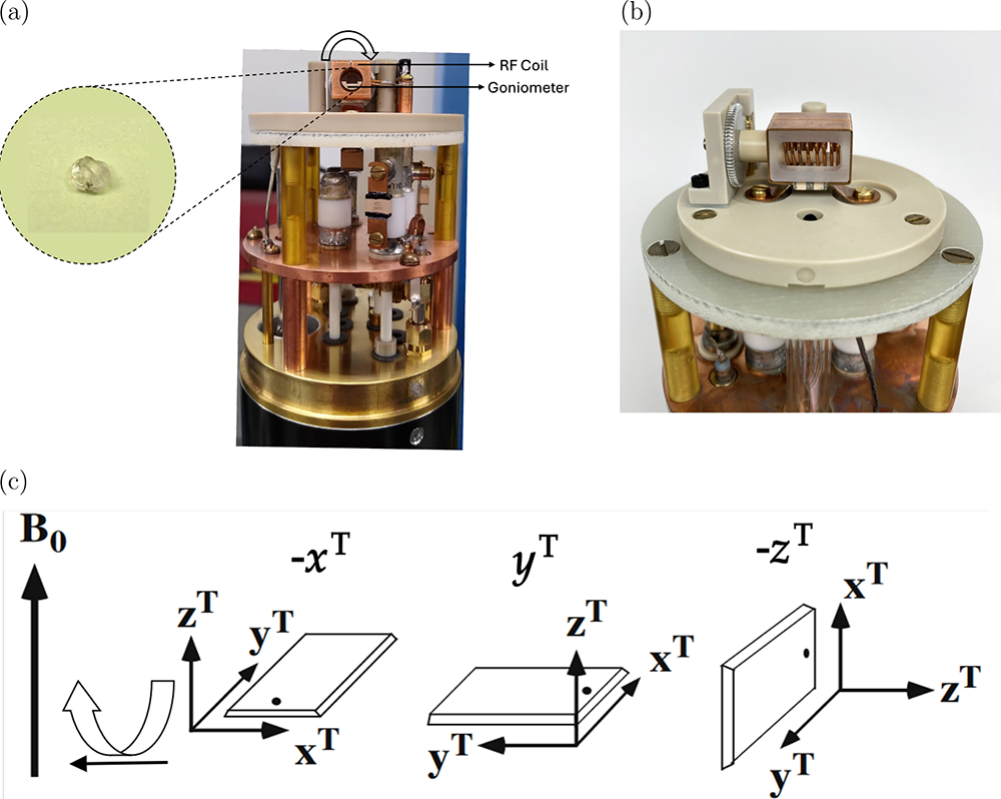
(a) Photograph of front view of the probe. The arrow shows rotation of the goniometer inside the RF coil. Inset shows l-alanine crystal glued to the tenon. The tenon may be mounted on three different orientations in the goniometer so that three orthogonal rotations of the sample can be achieved. (b) Photograph of side view of the probe showing goniometer mechanism and probe coils. (c) Rotation of tenon around an axis that is perpendicular to magnetic field. The three mutually perpendicular rotations are achieved by mounting the tenon plate into dovetails for −*x* ^*T*^, *y* ^*T*^, and −*z* ^*T*^ rotations. (This figure is modified from ref 30 under CC-BY 4.0 license, copyright T. Vosegaard.)

The CSA and quadrupolar tensor parameters *G*_mn_^ξ^ in the goniometer–tenon frame are determined by performing least-squares curve fitting of the experimentally acquired −*x* ^*T*^, *y* ^*T*^, and −*z* ^*T*^ rotation patterns using the expressions above.^32^ The tensor parameters in the respective principal axis frames (PAFs) of the CSA and quadrupolar tensors are then obtained via matrix diagonalization. The corresponding eigenvector matrices yield the relative orientations of each tensor with respect to the goniometer–tenon frame. These principal components represent unique molecular properties and provide a generalized framework for describing the corresponding NMR tensor interactions. In the principal axis frame (PAF), the CSA tensor can be represented using the Haeberlen convention^29^ as follows:

$$\delta_{\mathrm{iso}}=\frac{1}{3}\left(\delta_{11}+\delta_{22}+\delta_{33}\right)$$ 8

$$\delta_{\mathrm{CS}}=\delta_{33}-\delta_{\mathrm{iso}}$$ 9

$$\eta_{\mathrm{CS}}=\frac{\delta_{22}-\delta_{11}}{\delta_{\mathrm{CS}}}$$ 10

where

$$\left|\delta_{33}-\delta_{\mathrm{iso}}\right|\geq \left|\delta_{11}-\delta_{\mathrm{iso}}\right|\geq \left|\delta_{22}-\delta_{\mathrm{iso}}\right|$$ 11

The quadrupolar coupling constant (*C*_Q_) and asymmetry parameter (η_Q_) for electric field gradient (EFG) tensor can be expressed using the Haeberlen–Spiess convention^33^ as

$$C_{Q}=\frac{eQ\cdot V_{33}}{h}$$ 12

$$\eta_{Q}=\frac{V_{22}-V_{11}}{V_{33}}$$ 13

with

$$\left|V_{33}\right|\geq \left|V_{11}\right|\geq \left|V_{22}\right|$$ 14

The CSA and EFG tensors can be defined in their corresponding PAF using NMR parameters defined above (see Supporting Information).

Tensors defined in the principal axis frame (PAF) and the goniometer–tenon frame are connected via a unitary transformation. In most cases, the PAF of the quadrupolar interaction is chosen as the common reference frame, enabling expression of the CSA tensor’s PAF—–and when applicable, the crystal frame from X-ray diffraction—–in the same coordinate system. When the crystal frame is adopted as the common reference, both the quadrupolar and CSA PAFs can be mathematically converted into the goniometer–tenon frame, which corresponds to the physical orientation of the mounted crystal, using the following tensor transformations each consisting of three consecutive passive rotations involving an Euler’s angle set (α_1_, β_1_, γ_1_):

$$\mathrm{PAF}(\mathrm{CSA})\overset{\left(a,\;b,\;c\right)}{\to }\mathrm{PAF}(Q)\overset{\left(\zeta ,\;\lambda ,\;\nu \right)}{\to }\mathrm{Crystal}\;\mathrm{frame}\overset{\left(\alpha ,\;\beta ,\;\gamma \right)}{\to }\underset{\left(\mathrm{Rotating}\;\mathrm{frame}\right)}{\mathrm{Goniometer}\;\mathrm{frame}}$$ 15

Thus, the tensors in each frame, (e.g., A and B) are related by a unitary transformation as^34^

$$B=R(\alpha_{1},\;\beta_{1},\;\gamma_{1})AR^{-1}(\alpha_{1},\;\beta_{1},\;\gamma_{1})$$ 16

## Experimental Details

### ^17^O Labeling

The colorless d- and l- enantiomers of alanine were individually labeled by a saponification reaction of the corresponding alanine methyl ester hydrochloride (Ala-OMe·HCl) with sodium ethoxide (NaOEt) and isotopically enriched water (H_2_^17^O), following a procedure previously described.^15^ Both enantiomers of Ala-OMe·HCl and NaOEt were purchased from Sigma-Aldrich while H_2_^17^O with nominal 40% enrichment was purchased from Cambridge Isotope Laboratories (CIL). The relevant chemical reaction is given in Scheme 1. Details of the synthesis may be found in the Supporting Information. In this reaction, only one of the two carbonyl oxygens will originate from the labeled water reagent, so the product will have only half the starting atom-% label.

**Scheme 1:**
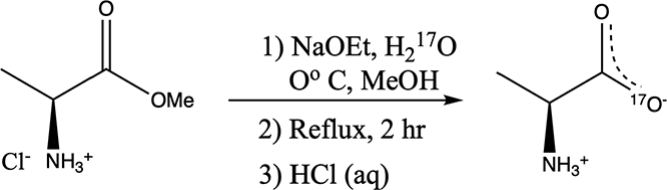
Synthesis of ^17^O-Labeled Alanine

Incorporation of the ^17^O label in the two alanine enantiomers was measured to be similar to the predicted 20 atom-% label by solution NMR, where the intensity of the ca. 265 ppm carboxylate resonance was referenced to that of the natural abundance ^17^O of the D_2_O solvent. Mass spectrometric analysis confirmed the isotope incorporation for each labeled compound (see Supporting Information). The ^1^H and ^13^C NMR spectra, all collected in D_2_O, were consistent with previous literature.^35^

Single crystals for both d- and l-alanine were prepared separately by slow evaporation at room temperature after dissolving ∼1.5 g of product in ∼9 mL cold deionized (DI) water. After about 45 days, crystals were harvested. The crystals were quickly washed with cold DI water to remove any surface impurities and stored at room temperature. Single crystals for the study were screened using the cross-polarized light microscopy technique^36^ to eliminate twinned and polycrystals; essentially, samples were observed while rotating them under crossed polarizers to verify uniform light transmission. The single crystals selected for the study had dimensions of approximately 4 × 5 × 2 mm^3^ for l-alanine and 3 × 5 × 2 mm^3^ for d-alanine. The crystals were glued to a tenon mounting plate for the goniometer NMR probe using epoxy resin.

### Single-Crystal X-ray Diffraction

The lattice structure parameters were determined using powder X-ray diffraction (XRD) analysis at the Electron Microbeam Analysis Laboratory (EMAL), University of Michigan. Powdered d- and l-alanine samples were analyzed in reflection mode in Bragg–Brentano geometry on a X-ray diffractometer (Rigaku Ultima IV). The Cu anode X-ray beam (40 kV, 44 mA) was filtered by a 20 μm thick nickel foil to remove Cu Kβ, giving monochromatic Cu Kα X-rays. The divergence, scattering and receiving slits were set at 2/3°, 2/3°, and 0.6 mm, respectively. The scanning 2θ range was from 5 to 70° with step size of 0.02° at a scan rate of 1° min^–1^. The unit cell parameters (*a*, *b*, *c*, α, β, γ) were recovered by assigning the appropriate triple of Miller indices (*hkl*) to each observed interplanar spacing (*d*_*hkl*_).The indexing process was performed using EXPO2014 ^37^ via the N-TREOR09 program,^38^ the evolution of the N-TREOR software.^39^ Powder XRD confirmed that the unit cells match the reported crystal structures;^26^ our measured d- and l-alanine crystal parameters are given in Table 1.

**Table 1:** Crystallographic Data for d-Alanine and l-Alanine at 295 K

	l-alanine	d-alanine
empirical formula	C_3_H_7_NO_2_	C_3_H_7_NO_2_
formula weight	75.08	75.08
crystal system	orthorhombic	orthorhombic
space group	*P*2_1_2_1_2_1_	*P*2_1_2_1_2_1_
*a*	6.036 (3) Å	6.025 (3) Å
*b*	12.342 (5) Å	12.324 (5) Å
*c*	5.788 (3) Å	5.783 (3) Å
*Z*	4	4
*Z*'	1	1
cell volume	431.2 Å^3^	429.4 Å^3^

The orientation of the mounted single crystals was determined using the so-called “Omega-Scan” method described elsewhere.^31^ The mounted crystal’s surface normal and edge plane directions were used to find the orientation matrix and ultimately the orientation of the crystal frames relative to the tenon frames. The Euler angles relating the crystal frame to the goniometer frame for the d-alanine crystal are 352.7, 90.0, 135.0° and for the l-alanine crystal are 333.5, 33.7, 90.0° (see Supporting Information for details).

### SCNMR Spectroscopy

NMR spectra for both samples were acquired using a Bruker Avance III console running Topspin 3.6 (Bruker Biospin GmbH) at the National High Magnetic Field Laboratory (NHMFL) in Tallahassee, FL. A custom-built low-E 600 MHz static HX probe (Figure 1a,b), developed at NHMFL, was used for the measurements. The probe features a cross-coil arrangement of a 6.5 mm ID round 9-turn X-channel detection solenoid coil mounted inside and orthogonal to a low inductance ^1^H-channel loop gap resonator was employed for the measurements. The low-E coil and associated probe circuitry have been thoroughly described in the literature.^40^

This probe was optimized for ^17^O detection with ^1^H decoupling and operated inside a 600 MHz (14.1 T), 89 mm bore magnet. The tenon plate, with the crystal glued on it, was mounted in the dovetail track of the goniometer and positioned within the 6 mm inner diameter loop-gap resonator-type NMR sample coil, enabling stepwise rotation to acquire SCNMR spectra across defined rotation patterns. The mounting configuration was designed to allow positive rotations of the tenon about the −*x* ^*T*^, *y* ^*T*^, and −*z* ^*T*^ axes, as defined by the dovetail pattern inscribed in the goniometer.^41^ Details of the goniometer mechanism and sample mount construction have been previously described.^31^ Figure 1 shows the probe, the single crystal sample mounted on the tenon, and the three orthogonal tenon rotations relative to the magnetic field, which vary depending on how the plate is mounted in the goniometer.

The mounted samples were rotated from 0 to 180° using the goniometer’s worm-gear mechanism, measured by adjusting an analog micrometer scale at the bottom of the probe. To minimize backlash error, all rotations were carried out in a single direction. The 90-degree pulse lengths were 4 μ*s* for the ^17^O channel and 2.5 μs for the ^1^H channel, respectively. Both the low frequency (^17^O) and high frequency (^1^H) channels were manually retuned and rematched at least every other angular increment. All spectra were acquired at room temperature (ca. 22 °C) with air cooling.

For each rotation axis, spectra were recorded with spectral width of 200 kHz and acquisition time of 0.02 s for l-alanine and spectral width of 100 kHz and acquisition time of 0.04 s for d-alanine. Between 256 and 512 transients were collected per spectrum, as needed to maintain a consistent signal-to-noise ratio (SNR). The rotation patterns were verified by observing smooth curves connecting the recorded resonance frequencies and by comparing the spectra at the starting and ending rotational positions, as well as key check points such as 0°(−*x* ^*T*^) = 0°(*y* ^*T*^), 90°(−*x* ^*T*^) = 90°(−*z* ^*T*^), and 0°(−*z* ^*T*^) = 90°(*y* ^*T*^). Spectral calibration was carried out using an external reference sample of tap water with the ^17^O chemical shift defined at 0.0 ppm.

### Computational Modeling

The literature neutron-diffraction crystal structures^26^ were used as the bases for the corresponding computed structures for l-alanine at 60 K (278467.cif) and 295 K (278464.cif) and for d-alanine at 60 K (278466.cif). The reference structure reported to be d-alanine at 295 K (278465.cif), was found to be the l-enantiomer, so a proxy for the d-enantiomer was created by inverting the *c*-axis of the l-alanine. The 60 K d-alanine structure has the N–C*–C angle oriented opposite those in the l-alanine structures, relative to the *a*-axis, so the literature structure was transformed by rotation about the *c*-axis to obtain equivalent computational starting points, where the only major differences between the two enantiomer structures was the relative orientation of the hydrogen and methyl groups on the chiral carbons (C*). All crystal operations were carried out using VESTA.^42^ Conversion of the neutron structure.cif files to the CASTEP.cell input files was completes using cif2cell.^43^

Although it is common practice to optimize experimental crystal structures prior to calculating magnetic parameters, we also calculated the electric field gradient (EFG) and chemical shielding tensors for the unoptimized structures to enable direct comparison with a previous literature report.^44^ These calculations were performed using both the rPBE functional^45^ with a 650 eV cutoff and the PBEsol functional with a 741 eV cutoff employing a 2 1 2 Monkhorst–Pack k-point grid^46^ in both cases (see Supporting Information).

The Electric Field Gradients^47^ (EFG) and chemical shielding tensors^28^ of both crystal enantiomers were computed through ultrasoft pseudopotentials^48,49^ using GIPAW (Gauge Including Projector Augmented Waves) approach,^28,50^ as implemented in the CASTEP-NMR package. The NMR parameters were then calculated using eqs 8–11 and eqs 12–14 and the mutual orientation of the two tensors was determined using eq 16.

For comparison, the EFG and shielding tensors were also calculated using optimized neutron structures (see Supporting Information). These calculations were conducted under 3D periodic boundary conditions using the CASTEP software package,^51^ employing the PBEsol exchange-correlation functional,^27,52,53^ and “precise” basis set precision with automatic finite-basis-set correction^54^ and Tkatchenko-Scheffler semiempirical dispersion correction.^55^ The planewave cutoff energy was 741 eV, and a 2 1 2 Monkhorst–Pack k-point grid was again used. During optimization, unit-cell parameters were fixed to their literature values, while atomic positions were allowed to relax.^56–58^ The minor differences between the experimental and optimized structures are summarized as root-mean-square displacements in Table 2.

**Table 2:** Average Root-Mean-Square Deviations in Nuclear Positions after Optimization of l- and d-Alanine from Literature Crystal Structures

		RMS deviation/10^–4^ Å			
nucleus	# centers	l-ala (295 K)	d-ala (295 K)^a^	l-ala (60 K)	d-ala (60 K)
H	28	2.48	2.50	1.72	1.10
C	12	5.53	5.60	3.21	5.16
N	4	1.54	1.47	1.59	0.63
O	8	9.23	7.70	9.73	9.36
All	52	4.87	4.46	4.33	4.51

a Starting structure derived from l-alanine (295 K).

## Results and Discussion

### Crystal Structure

As shown in Figure 2, the unit cell for alanine has two nonequivalent carboxylate O sites in each of the four molecules of the unit cell, related by screw symmetry, leading to four magnetically nonequivalent O nuclei in each site. As a result, an independent resonance frequency is expected from each of the magnetically nonequivalent O nuclei.

**Figure 2:**
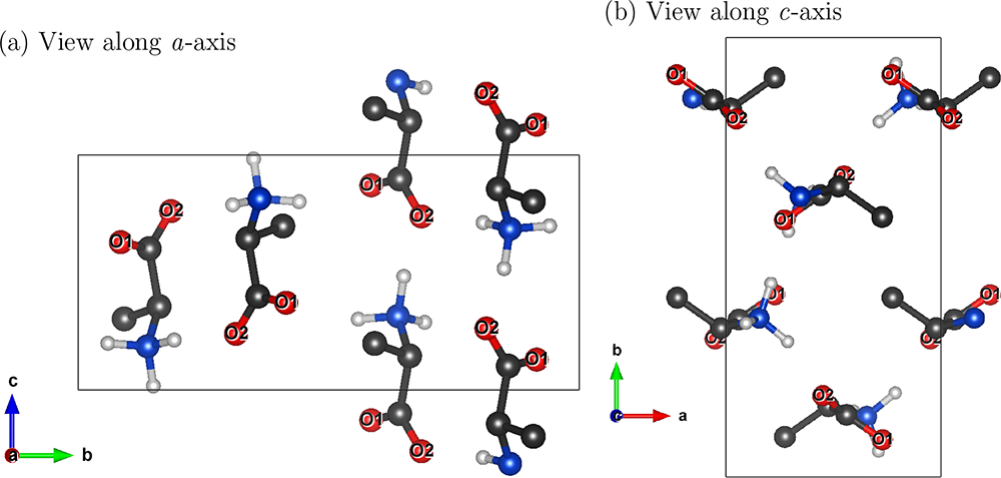
(a, b) Two views of the orthorhombic crystal structure of l-alanine the crystallographically nonequivalent O sites, related by a screw axis, labeled as O1 and O2. All the eight O nuclei are magnetically nonequivalent. The arrows represent directions and scaled magnitudes of the computed chemical shielding tensors in the crystal frame. O, C, N, and H nuclei are shown in red, black, blue, and white, respectively. (H nuclei on carbons omitted for clarity.)

### Single-Crystal ^17^O NMR

The single-crystal ^17^O NMR spectra for l-alanine rotation about −*x* ^*T*^, *y* ^*T*^, and −*z* ^*T*^ axes are shown in Figure 3 and those for d-alanine in Figure 4. A maximum of eight equally intense transitions for each orientation of the single crystal were observed, as expected from eight magnetically nonequivalent O sites. While we were able to observe the satellite transitions (see Supporting Information), limited time and resources prevented us from measuring the systematic shift of satellite transitions required to extract the quadrupolar ACS cross-coupling terms. We believe that differences in the signal-to-noise intensity between angles were related to relative sample filling factor.

**Figure 3:**
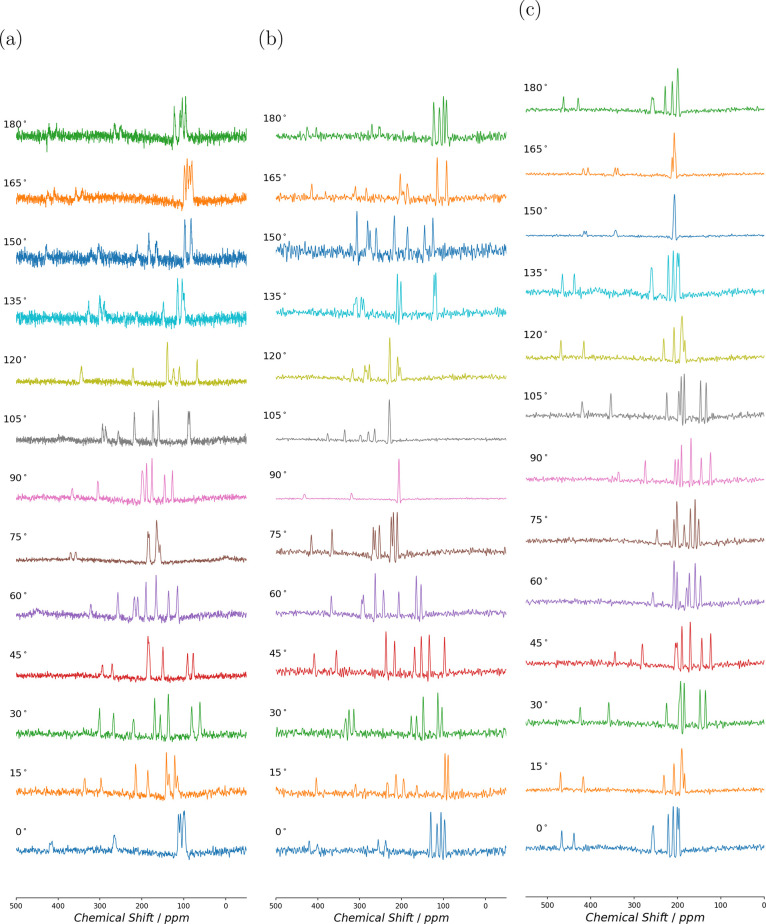
Single-crystal ^17^O NMR spectra at 14.1 T for l-alanine. The spectra were recorded at 15° increments for rotation about (a) mounting −*x* ^*T*^, (b) mounting *y* ^*T*^, and (c) mounting −*z* ^*T*^.

**Figure 4:**
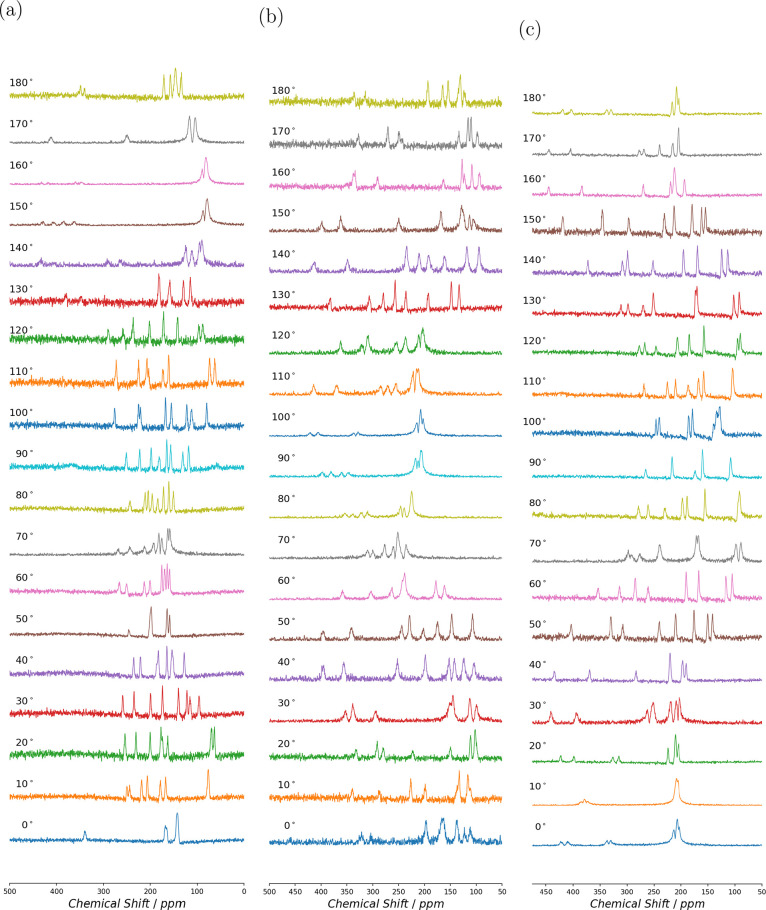
Single-crystal ^17^O NMR spectra at 14.1 T for d-alanine. The spectra were recorded at 10° increments for rotation about (a) mounting −*x* ^*T*^, (b) mounting *y* ^*T*^, and (c) mounting −*z* ^*T*^.

The correlations of the rotation plots and peak assignments was assisted by the fact that the NMR chemical shift frequency of each site remains the same for certain pairs of crystal orientations (eqs 17–19), where the mountings are as shown in Figure 1c. The pairwise correlation is not obvious from the spectra shown in Figures 3 and 4, since the crystal axes are not generally aligned with the tenon axes, but it may be observed in the rotation plots in Figures 5 and 6. This pairwise coincidence results from NMR interactions being insensitive to rotation parallel to the magnetic field axis.^59^

$$\mathrm{Mounting}\;-x^{T}\;(\Theta =0^{{}^{\circ}})≡\mathrm{Mounting}\;y^{T}\;(\Theta =0^{{}^{\circ}})$$ 17

$$\mathrm{Mounting}\;-x^{T}\;(\Theta =90^{{}^{\circ}})≡\mathrm{Mounting}\;-z^{T}\;(\Theta =90^{{}^{\circ}})$$ 18

$$\mathrm{Mounting}\;y^{T}\;(\Theta =90^{{}^{\circ}})≡\mathrm{Mounting}\;-z^{T}\;(\Theta =0^{{}^{\circ}})$$ 19

**Figure 5:**
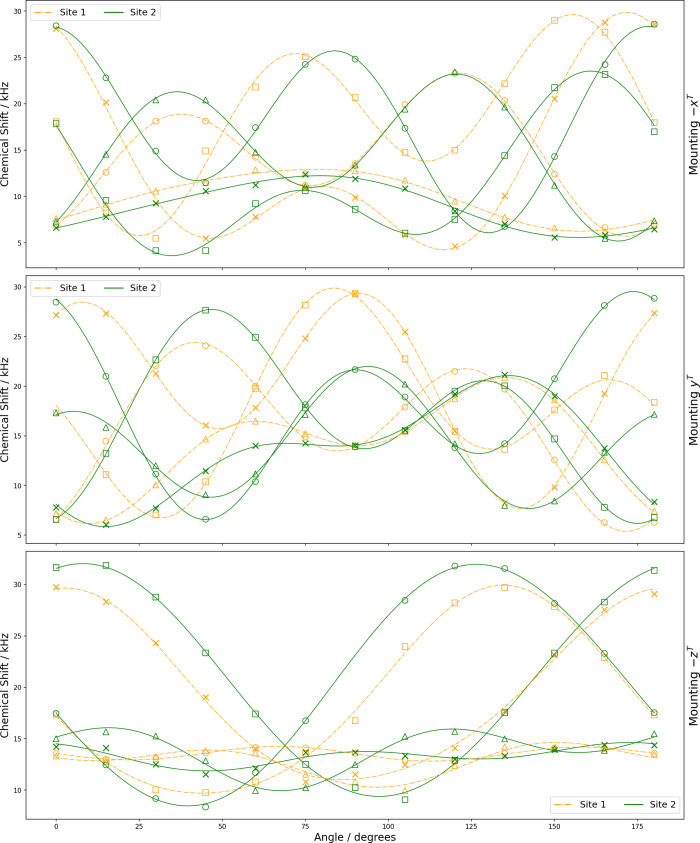
Rotation plots for ^17^O central transition in l-alanine showing experimental resonances with two crystallographically nonequivalent ^17^O sites each having four magnetically nonequivalent ^17^O nuclei (marked as ○, □, △, ×) under rotation of the three orientations of the crystal sample. The curves are constructed from optimized coefficients (see Table 3).

**Figure 6:**
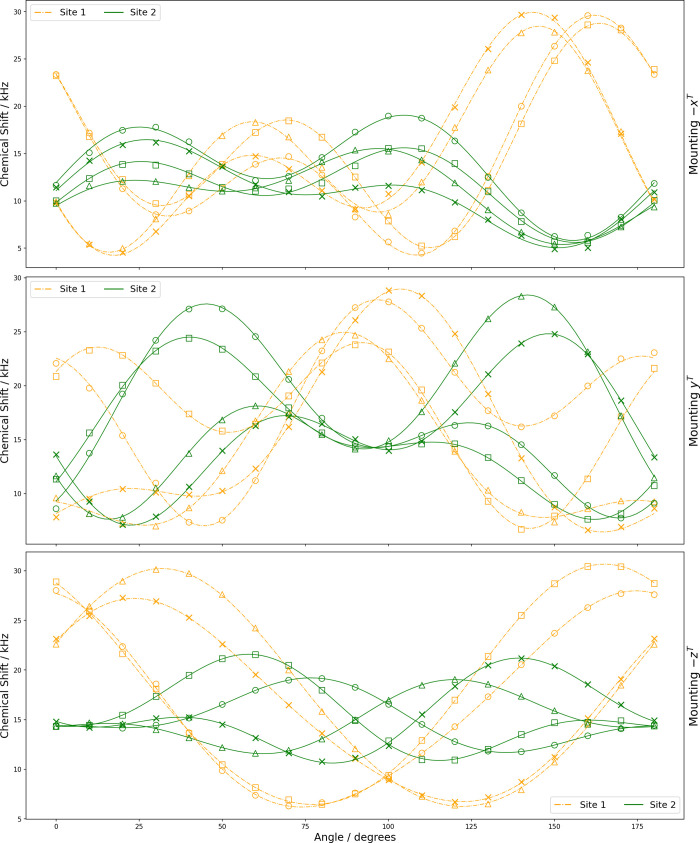
Rotation plots for ^17^O central transition in d-alanine showing experimental resonances with two crystallographically nonequivalent ^17^O sites each having four magnetically nonequivalent ^17^O nuclei (marked as ○, □, △, ×) under rotation of the three orientations of the crystal sample. The curves are constructed from optimized coefficients (see Table 4).

### Analysis of SCNMR Spectra

#### ^17^O NMR Parameters

The optimized quadrupolar and CSA parameters, along with their errors, were obtained by fitting the central transition of each site using the Analysis of Single-Crystal Spectra (*ASICS*) software package.^59^ The rotation plots shown in Figures 5 and 6 were fit according to eqs 6 and 7. Tables 3 and 4 summarize the optimized coefficients provided by *ASICS* for magnetically nonequivalent O nuclei in l-alanine and d-alanine.

**Table 3:** Optimized Coefficients (in kHz) for ^17^O Rotation Data of l-Alanine (up to 3 Significant Figures)

mounting	nucleus	*A*	*B*	*C*	*D*	*E*
–*x* ^*T*^	1	14.7	–3.37	–1.05	–4.51	4.25
*y* ^*T*^		20.2	–1.06	3.90	8.08	2.59
–*z* ^*T*^		20.6	10.9	2.99	0.0857	0.151
–*x* ^*T*^	2	15.1	–3.05	0.266	–4.81	5.09
*y* ^*T*^		17.8	3.54	–3.81	7.41	–1.81
–*z* ^*T*^		19.4	9.26	0.309	0.972	0.523
–*x* ^*T*^	3	17.9	1.69	2.62	8.68	–2.39
*y* ^*T*^		13.7	–2.99	–4.76	–2.61	–1.80
–*z* ^*T*^		18.6	–0.238	–10.1	–1.17	0.00316
–*x* ^*T*^	4	11.4	4.52	–4.75	1.77	–5.42
*y* ^*T*^		14.2	–3.35	–3.13	–3.54	–1.75
–*z* ^*T*^		20.2	–2.81	–11.4	0.125	0.289
–*x* ^*T*^	5	18.6	–1.19	–4.39	0.646	–8.69
*y* ^*T*^		17.0	–3.58	3.85	–6.80	0.814
–*z* ^*T*^		13.8	1.36	–1.27	0.00303	2.00
–*x* ^*T*^	6	13.5	9.16	–2.22	5.72	–4.05
*y* ^*T*^		16.1	–3.60	2.31	–5.84	3.21
–*z* ^*T*^		13.3	0.387	–0.628	0.812	–0.167
–*x* ^*T*^	7	9.90	–2.60	2.00	0.187	0.391
*y* ^*T*^		17.8	–5.53	–1.83	5.76	–4.37
–*z* ^*T*^		13.5	0.0178	0.0635	0.00522	–0.667
–*x* ^*T*^	8	8.95	–2.69	1.82	0.333	0.188
*y* ^*T*^		14.0	–2.32	0.210	5.41	1.76
–*z* ^*T*^		12.8	1.35	0.0144	–1.04	–0.714

**Table 4:** Optimized Coefficients (in kHz) for ^17^O Rotation Data of d-Alanine (up to 3 Significant Figures)

mounting	nucleus	*A*	*B*	*C*	*D*	*E*
–*x* ^*T*^	1	14.7	7.22	–3.12	1.49	–7.19
*y* ^*T*^		16.8	–2.66	5.99	–4.81	1.26
*–z* ^*T*^		17.7	5.26	10.8	–0.297	0.450
*–x* ^*T*^	2	15.6	5.46	–1.24	2.31	–7.60
*y* ^*T*^		15.5	–1.64	5.97	–2.77	2.32
*–z* ^*T*^		16.4	5.96	8.18	0.555	0.773
*–x* ^*T*^	3	15.2	0.0720	–5.50	–5.49	–5.99
*y* ^*T*^		17.3	–1.32	4.26	5.32	2.95
*–z* ^*T*^		17.9	10.7	–5.69	0.159	–0.638
*–x* ^*T*^	4	15.0	0.311	–8.01	–5.37	–4.87
*y* ^*T*^		18.4	–2.32	–4.82	6.52	0.922
*–z* ^*T*^		16.5	10.1	–3.54	1.14	0.382
*–x* ^*T*^	5	13.6	–2.57	2.12	0.876	4.43
*y* ^*T*^		17.2	–1.31	–6.04	–4.35	–3.78
*–z* ^*T*^		15.6	–0.358	3.83	–0.928	–2.24
*–x* ^*T*^	6	11.4	–2.15	1.44	0.601	3.22
*y* ^*T*^		15.8	–0.694	–5.21	–1.64	–4.58
*–z* ^*T*^		15.0	–1.94	2.06	1.29	–0.925
*–x* ^*T*^	7	10.9	–2.86	1.726	1.46	2.08
*y* ^*T*^		15.1	–8.97	–3.20	2.06	4.02
*–z* ^*T*^		15.0	–0.337	–2.65	–0.337	1.64
*–x* ^*T*^	8	10.9	–0.0933	3.80	0.263	2.75
*y* ^*T*^		13.3	–7.79	0.598	3.76	–0.998
*–z* ^*T*^		15.5	1.93	–3.02	–2.57	–0.0608

Table 5 summarizes the optimized experimental parameters, with error limit estimated as 95% confidence intervals for individual parameters, along with the DFT computations of quadrupolar coupling and CSA parameters. Typical standard deviations in the fitted experimental peaks were 0.063 ppm for l-alanine and 0.029 ppm for d-alanine. The error bars for the derived experimental parameters in Table 5 are determined using the ASICS data-fitting program. In the “Analysis” mode, the parameters may be scanned while monitoring deviation of the fitted data. In most cases, this was a symmetric quadratic, from which the 95% confidence intervals were extracted. In *ASICS*, the NMR parameters are refined according to the minimum χ^2^ (goodness of fit) deviation. The χ^2^ distribution is also used to find the 95% confidence interval^59^ for the parameters. For two parameters, the confidence interval could not be estimated as χ^2^ values for those parameters were not distributed as a quadratic function and did not converge to a minimum. The two anionic oxygen sites in the zwitterionic alanine crystal have different orientations relative to the cationic ammonium moieties and thus different hydrogen bonding interactions, resulting in distinct magnetic environments, they have, consequently, distinct NMR parameters.

**Table 5:** Experimental and Computed Quadrupolar Couplings, Chemical Shift Anisotropies, Isotropic Chemical Shift, and Relative Orientations of the Two Tensors for ^17^O and ^14^N Nuclei in l-Alanine and d-Alanine

compound	nucleus	site	|*C*_Q_|/MHz	η_Q_	δ_CS_/ppm	η_CS_	δ_iso_ ^a^/ppm	*a*/°	*b*/°	*c*/°	reference	method
experimental												
l-alanine	^17^O	1	6.5 ± 0.4	0.34 ± 0.16	173 ± 27	0.4 ± 0.4	227^b^	2.3^b^	86 ± 3	92 ± 14	this study	SCNMR
		2	6.6 ± 0.4	0.63 ± 0.17	189 ± 22	0.5 ± 0.2	238.8 ± 7.8	32 ± 16	86 ± 3	89 ± 5		
	^17^O	1	7.86 ± 0.05	0.28 ± 0.02			284.0 ± 0.5				^24^	MAS
		2	6.53 ± 0.05	0.70 ± 0.02			260.5 ± 0.5					
	^17^O	1	8.1 ± 0.3				285 ± 8				^23,24^	DAS
		2	7.2 ± 0.3				268 ± 8					
	^14^N		1.14 ± 0.03	0.24 ± 0.03			3.1 ± 0.3				^60^	MAS
d-alanine	^17^O	1	7.1 ± 0.2	0.24 ± 0.09	188 ± 15	0.4 ± 0.12	235.5 ± 4.9	24 ± 10	89.1 ± 1.5	86 ± 10	this study	SCNMR
		2	5.91 ± 0.13	0.70 ± 0.06	153 ± 7	0.83 ± 0.09	224.8 ± 2.8	27 ± 3	88.1 ± 1.0	91.0 ± 1.3		
	^17^O	1	7.60 ± 0.02	0.60 ± 0.01			275 ± 5				^25^	MQMAS
		2	6.40 ± 0.02	0.65 ± 0.01			262 ± 5					
calculated												
PBEsol + GIPAW												
l- and d-alanine	^17^O	1	8.09	0.22	282	0.48	– 30.7^c^	39	89	97	this study	
		2	6.49	0.65	202	0.68	– 11.9^c^	29	88	95		
	^14^N		1.29	0.25	10.4	0.99	186.4^c^	125	150	150	this study	

a Isotropic chemical shifts are relative to tap water at 0.0 ppm.

b Error limit could not be estimated for the parameter.

c Reported values are σ_iso_.

Overall, we observed good correlation between the experimental and calculated values for the two enantiomers of alanine. However, some discrepancies remain between the experimental and calculated NMR parameters, even within the 95% confidence interval. In particular, the comparison reveals disagreement in *C*_Q_ and δ_CS_ for at least one site. For *C*_Q_, the largest deviation occurs at site O1, where the calculated value is approximately 24% higher for the l-enantiomer and about 14% higher for the d-enantiomer. A similar trend has been reported in previous ^17^O studies of amino acid samples.^61^

In the case of δ_CS_, the asymmetry parameter of the CSA tensor, the largest discrepancy is again observed at site O1, with calculated values 63% higher for l-alanine and 50% higher for d-alanine. Since NMR parameters are sensitive to the chemical environments, the weaker H-bonding environment at site O1—involved in only one C=O···H–N hydrogen bond^25^—likely contributes to the observed differences between the experimental and DFT-calculated values.^62^

Table 5 also presents values of *C*_Q_, η_Q_, and δ_iso_ from previous experimental studies using powder samples. The SCNMR experimental η_*Q*_ values obtained from this study show good agreement with the MAS^24^ and DAS^23^ measurements. However, there is notable disagreement with the MQMAS measurements^25^ for d-alanine, though the MQMAS measurements also differ from other studies as well as from the computational findings in this study. In contrast, the DFT calculated NMR parameters reported here are in good agreement with most of the experimental results.

The *C*_Q_ and δ_iso_ have been identified as reliable parameters to assign sites O1 and O2.^25^ Given the nearly identical and relatively large uncertainties in experimental *C*_Q_ values, and our inability to determine 95% confidence interval for δ_iso_ in one of the two l-alanine sites, we have turned to the asymmetry parameter, η_Q_, for the site assignment. This parameter is particularly useful, as it is known to increase with the strength of hydrogen bonding.^61^

The Euler angles *a*, *b*, and *c* agree well for both enantiomers when compared with the calculated values. Specifically, the angles *b* and *c* align well with the computed value for both enantiomers. The other angle, *a* shows the largest statistical uncertainty, which is related to the inaccurate measurement of η_CS_,^32^ with experimental η_CS_ values having variation between 11 and 100% in statistical uncertainty. Due to the potential ambiguity in the projection direction of the quadrupolar and CSA principal axes, the Euler angles have been rotated by 180° where necessary to allow direct comparison. The Euler angles (ζ, λ, ν) relating the principal axis frame of quadrupolar tensor (PAF­(Q)) to crystal axis frame were calculated using the relationship

$$R(\zeta ,\;\lambda ,\;\nu )=R^{-1}(\alpha ,\;\beta ,\;\gamma )R(\alpha_{\mathrm{QG}},\;\beta_{\mathrm{QG}},\;\gamma_{\mathrm{QG}})$$

where the Euler triplet (α, β, γ) relates crystal axis frame with goniometer frame and was found using X-ray diffraction. The Euler triplet (α_QG_, β_QG_, γ_QG_) gives the orientation of PAF­(Q) to goniometer frame. Both sets of Euler angles are reported in Tables 6 and 7.

**Table 6:** Experimental Euler Angles Relating the PAF­(Q) to Goniometer and Crystal Axis Frame for Magnetically Equivalent ^17^O Nuclei for Two Crystallographically Nonequivalent Sites in l-Alanine

site	nucleus	α_QG_	β_QG_	γ_QG_	ζ	λ	ν
1	1	48	47	336	82	66	73
	2	310	24	102	–12	11	–1
	3	129	50	203	–85	68	–22
	4	118	7	58	79	28	34
2	1	263	9	150	–24	30	11
	2	223	1	2	–46	34	28
	3	170	38	323	20	64	60
	4	145	56	38	6	42	–54

**Table 7:** Experimental Euler Angles Relating the PAF­(Q) to Goniometer and Crystal Axis Frame for Magnetically Equivalent ^17^O Nuclei for Two Crystallographically Nonequivalent Sites in d-Alanine

site	nucleus	α_QG_	β_QG_	γ_QG_	ζ	λ	ν
1	1	304	21	80	67	78	25
	2	308	19	80	71	79	23
	3	248	35	80	8	71	37
	4	–90	39	70	20	75	44
2	1	318	39	348	–2	122	31
	2	210	19	19	–84	98	25
	3	37	36	180	88	66	–20
	4	342	66	40	70	94	73

In addition to the conventional CSA and quadrupolar tensor values, our DFT calculations also yielded the antisymmetric components of the chemical shift tensors for both ^17^O and ^14^N nuclei, as shown in Tables 8–10. These tables reveal that the antisymmetric chemical shift tensor components of ^17^O and ^14^N nuclei exhibit relationships such as σ_*xz*_^ACS^(*L*) = −σ_*xz*_^ACS^(*R*) and σ_*yz*_^ACS^(*L*) = −σ_*yz*_^ACS^(*R*) between the two enantiomers. This confirms the mirror reflection symmetry relating the electronic environments of the two enantiomers across the xy plane. To depict the computed ACS tensors for the alanine enantiomers in their respective crystal frames, we employ the pseudovector components resulting from the contraction of ACS tensor σ^ACS^ and Levi-Civita symbol ε_*ijk*_ according to eqs 20 and 21:^63^

$$V_{i}=\varepsilon_{ijk}\sigma_{jk}^{\mathrm{ACS}}$$ 20

giving,

$$\begin{array}{ccc} V_{x}=2\sigma_{yz}^{\mathrm{ACS}}, & V_{y}=2\sigma_{zx}^{\mathrm{ACS}}, & V_{z}=2\sigma_{xy}^{\mathrm{ACS}} \end{array}$$ 21

Figure 7 shows the pseudovectors that are dual of the DFT computed ACS tensors for sites O1 and O2, highlighting the mirror plane symmetries of the l- and d-enantiomers, and demonstrating the antisymmetry of the ACS tensors in chiral systems. The calculated tensors are given in Tables 8 and 9.

**Table 8:** Calculated quadrupolar (in MHz) and Chemical Shielding (CS) Tensors (in ppm), Rounded to Three Decimal Places, for One of the Magnetically Equivalent ^17^O Site for Crystallographic Site O1 in Enantiomers of Alanine

	enantiomer	
component	l-alanine	d-alanine
quadrupolar	$$\left(\begin{array}{ccc} -4.120 & 0.726 & -1.187\\ 0.726 & -3.623 & 1.702\\ -1.187 & 1.702 & 7.743 \end{array}\right)$$	$$\left(\begin{array}{ccc} -4.120 & 0.726 & 1.187\\ 0.726 & -3.623 & -1.702\\ 1.187 & -1.702 & 7.743 \end{array}\right)$$
total CS	$$\left(\begin{array}{ccc} -51.128 & 209.778 & 55.794\\ 180.674 & 120.849 & -83.964\\ 19.534 & -46.887 & -161.699 \end{array}\right)$$	$$\left(\begin{array}{ccc} -51.139 & 209.781 & -55.794\\ 180.676 & 120.751 & 83.968\\ -19.532 & 46.886 & -161.799 \end{array}\right)$$
isotropic CS	$$\left(\begin{array}{ccc} -30.659 & 0 & 0\\ 0 & -30.659 & 0\\ 0 & 0 & -30.659 \end{array}\right)$$	$$\left(\begin{array}{ccc} -30.729 & 0 & 0\\ 0 & -30.729 & 0\\ 0 & 0 & -30.729 \end{array}\right)$$
symmetric CS	$$\left(\begin{array}{ccc} -51.128 & 195.226 & 37.664\\ 195.226 & 120.849 & -65.425\\ 37.664 & -65.425 & -161.699 \end{array}\right)$$	$$\left(\begin{array}{ccc} -51.139 & 195.229 & -37.663\\ 195.229 & 120.751 & 65.427\\ -37.663 & 65.427 & -161.799 \end{array}\right)$$
antisymmetric CS	$$\left(\begin{array}{ccc} 0 & 14.552 & 18.130\\ -14.552 & 0 & -18.539\\ -18.130 & 18.539 & 0 \end{array}\right)$$	$$\left(\begin{array}{ccc} 0 & 14.553 & -18.131\\ -14.553 & 0 & 18.541\\ 18.131 & -18.541 & 0 \end{array}\right)$$

**Table 9:** Calculated quadrupolar (in MHz) and Chemical Shielding (CS) Tensors (in ppm), Rounded to Three Decimal Places, for One of the Magnetically Equivalent ^17^O Site for Crystallographic Site O2 in Enantiomers of Alanine

	enantiomer	
component	l-alanine	d-alanine
quadrupolar	$$\left(\begin{array}{ccc} 0.942 & -1.012 & 4.634\\ -1.012 & -0.817 & -3.580\\ 4.634 & -3.580 & -0.125 \end{array}\right)$$	$$\left(\begin{array}{ccc} 0.942 & -1.012 & -4.634\\ -1.012 & -0.817 & 3.580\\ -4.634 & 3.580 & -0.125 \end{array}\right)$$
total CS	$$\left(\begin{array}{ccc} -63.835 & 165.365 & -76.405\\ 168.444 & 77.812 & 26.025\\ 7.265 & -23.272 & -49.805 \end{array}\right)$$	$$\left(\begin{array}{ccc} -63.854 & 165.377 & 76.405\\ 168.449 & 77.714 & -26.028\\ -7.269 & 23.276 & -49.924 \end{array}\right)$$
isotropic CS	$$\left(\begin{array}{ccc} -11.943 & 0 & 0\\ 0 & -11.943 & 0\\ 0 & 0 & -11.943 \end{array}\right)$$	$$\left(\begin{array}{ccc} -12.021 & 0 & 0\\ 0 & -12.021 & 0\\ 0 & 0 & -12.021 \end{array}\right)$$
symmetric CS	$$\left(\begin{array}{ccc} -63.835 & 166.905 & -34.57\\ 166.905 & 77.812 & 1.376\\ -34.570 & 1.376 & -49.805 \end{array}\right)$$	$$\left(\begin{array}{ccc} -63.854 & 166.913 & 34.568\\ 166.913 & 77.714 & -1.376\\ 34.568 & -1.376 & -49.924 \end{array}\right)$$
antisymmetric CS	$$\left(\begin{array}{ccc} 0 & -1.539 & -41.835\\ 1.539 & 0 & 24.648\\ 41.835 & -24.648 & 0 \end{array}\right)$$	$$\left(\begin{array}{ccc} 0 & -1.536 & 41.837\\ 1.536 & 0 & -24.652\\ -41.837 & 24.652 & 0 \end{array}\right)$$

**Table 10:** Calculated quadrupolar (in MHz) and Chemical Shielding (CS) Tensors (in ppm), Rounded to Three Decimal Places, for One of the Magnetically Equivalent ^14^N Site in Enantiomers of Alanine

	enantiomer	
component	l-alanine	d-alanine
quadrupolar	$$\left(\begin{array}{ccc} 0.433 & -0.236 & -0.975\\ -0.236 & -0.421 & 0.283\\ -0.975 & 0.283 & -0.012 \end{array}\right)$$	$$\left(\begin{array}{ccc} 0.433 & -0.236 & 0.975\\ -0.236 & -0.421 & -0.283\\ 0.975 & -0.283 & -0.012 \end{array}\right)$$
total CS	$$\left(\begin{array}{ccc} 188.770 & 1.781 & -4.136\\ -1.286 & 176.790 & -2.976\\ -4.771 & -3.925 & 193.611 \end{array}\right)$$	$$\left(\begin{array}{ccc} 188.766 & 1.779 & 4.136\\ -1.286 & 176.696 & 2.980\\ 4.770 & 3.925 & 193.518 \end{array}\right)$$
isotropic CS	$$\left(\begin{array}{ccc} 186.390 & 0 & 0\\ 0 & 186.390 & 0\\ 0 & 0 & 186.390 \end{array}\right)$$	$$\left(\begin{array}{ccc} 186.327 & 0 & 0\\ 0 & 186.327 & 0\\ 0 & 0 & 186.327 \end{array}\right)$$
symmetric CS	$$\left(\begin{array}{ccc} 188.770 & 0.248 & -4.453\\ 0.248 & 176.790 & -3.450\\ -4.453 & -3.450 & 193.611 \end{array}\right)$$	$$\left(\begin{array}{ccc} 188.766 & 0.246 & 4.453\\ 0.246 & 176.696 & 3.453\\ 4.453 & 3.453 & 193.518 \end{array}\right)$$
antisymmetric CS	$$\left(\begin{array}{ccc} 0 & 1.534 & 0.318\\ -1.534 & 0 & 0.474\\ -0.318 & -0.474 & 0 \end{array}\right)$$	$$\left(\begin{array}{ccc} 0 & 1.532 & -0.317\\ -1.532 & 0 & -0.472\\ 0.317 & 0.472 & 0 \end{array}\right)$$

**Figure 7:**
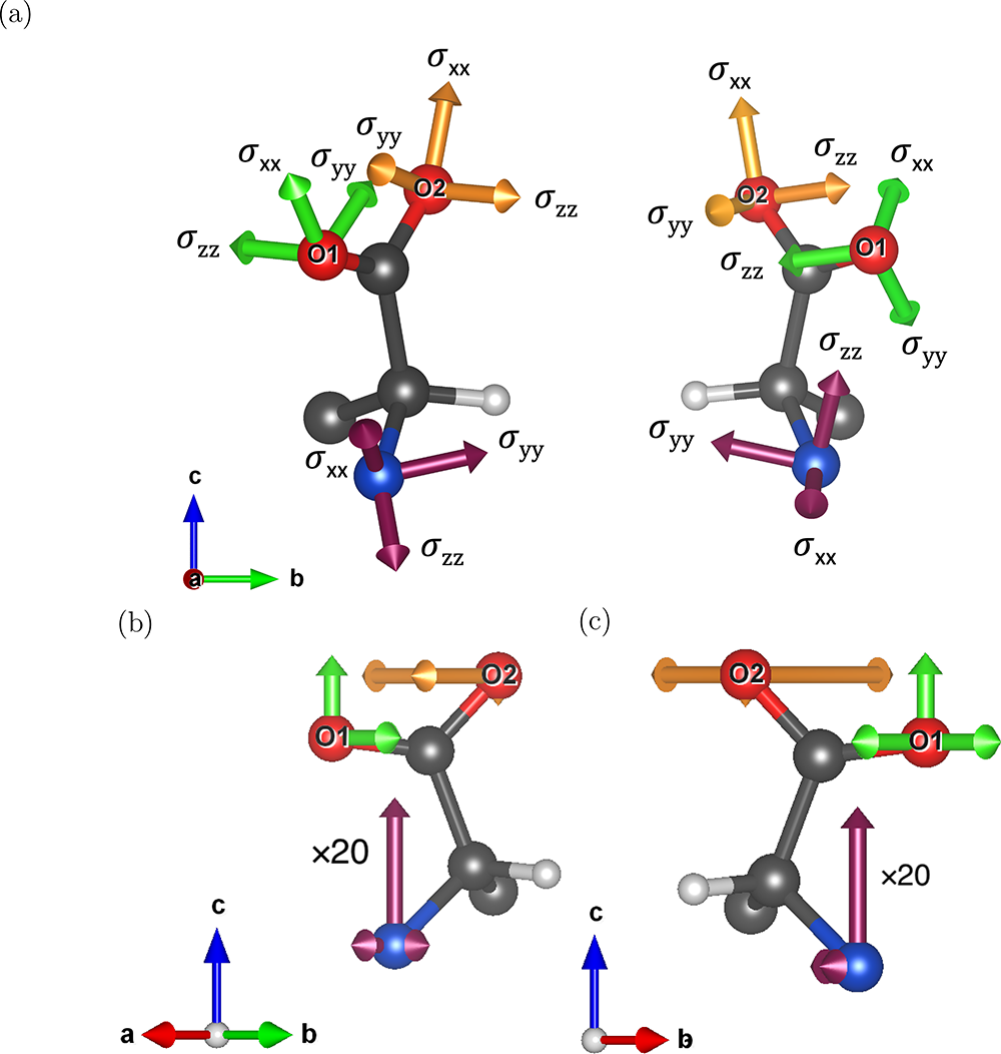
Depictions of (**top**) the shielding tensor eigenvectors and (**bottom**) the ACS pseudovectors for the O1 and O2 oxygen sites, and the nitrogen site in (**left**) l-alanine and (**right**) d-alanine, where *V*_*x*_ ∥ *a*, *V*_*y*_ ∥ *b*, and *V*_*z*_ ∥ *c*. The tensors at the N site are scaled by a factor of 20 so they are visible on the same scale as the O tensors. The coordinate axes in the pseudovector depiction have been rotated so that the structural symmetry is emphasized. Note that the *V*_*x*_ pseudovector on O1 is obscured by the atom and the *V*_*z*_ pseudovector on O2 is very small. C, N, O, and H atoms are shown in black, blue, red, and white, respectively. All hydrogens, except that on the chiral carbon, have been omitted for clarity.

#### ^14^N NMR Parameters

Unfortunately, our efforts to observe the low-sensitivity ^14^N nucleus in chiral systems have thus far been unsuccessful, although we were able to detect ^14^N in the highly symmetric environment of ammonium chloride. The ^14^N nucleus (*I* = 1, γ_^14^N_ = 1.934 × 10^7^ rad T^–1^ s^–1^) has a low gyromagnetic ratio, two nonsymmetrical satellite transitions (|1⟩↔|0⟩ and |0⟩↔|−1⟩), and a relatively large quadrupolar coupling constant (*C*_Q_). These factors contribute to line broadening and signal loss, making ^14^N in an asymmetric environment particularly challenging to detect.^44,64^ However, the good correlation for ^17^O between experimental and DFT results provides some confidence for the calculated tensors for ^14^N given in Table 10, particularly given the good agreement between calculated and experimental *C*_Q_ and η_Q_ values.^60^ The chemical shielding eigenvectors and ACS pseudovectors for the N site are also depicted in Figure 7.

## Conclusions

In this study, we investigated the quadrupolar and chemical shielding tensor components of ^17^O-enriched alanine enantiomers using single-crystal ssNMR spectroscopy. Eight magnetically inequivalent ^17^O sites, as predicted by X-ray crystallography, were successfully identified and analyzed for their NMR tensor parameters. The experimental analysis performed using the *ASICS* software package, as described in the Analysis of SCNMR Spectra section, was further supported by DFT calculations. The DFT calculations not only showed good correlation with experimental NMR parameters but also aided in assigning the crystallographically nonequivalent ^17^O sites, thereby validating the spectroscopic findings.

This study provides, for the first time, an extended and detailed characterization of the crystallographically distinct ^17^O nuclei in alanine enantiomers. The obtained NMR parameters are in good agreement with previous studies, reinforcing the robustness of our approach. Although the antisymmetric chemical shift (ACS) tensor components could not be directly extracted from the ssNMR experiments—since only the central transition of the spin-5/2 ^17^O nucleus was analyzed, which lacks ACS contributions—the DFT results revealed the presence of off-diagonal ACS elements (e.g., σ_*yz*_^ACS^ = −σ_*zy*_^ACS^) in the crystal frame. These components reflect the mirror-symmetric electronic environments characteristic of the optical isomers.

Our findings demonstrate the utility of combining SCNMR spectroscopy with DFT calculations to discern subtle differences in electronic environments between enantiomers. This capability underscores the power of SCNMR for probing chiral molecular systems such as amino acids. Future studies may focus on analyzing all satellite transitions of ^17^O nuclei in chiral environments to experimentally access ACS contributions through Quadrupolar–ACS interactions.^19^ Such interactions can produce observable features in the angular-dependent rotation patterns of single-crystal ssNMR spectra, in addition to the conventional first- and second-order quadrupolar and CSA effects. Furthermore, we propose extending this methodology to ^14^N SCNMR at higher magnetic fields, where enhanced sensitivity and resolution may enable clearer observation of quadrupolar broadening and magnetic shielding anisotropy. These studies could provide deeper insights into antisymmetric shielding effects and their potential role in chiral selection mechanisms, offering a plausible explanation for the enantiomeric excess of l-amino acids observed in carbonaceous chondrite meteorites—and thus contributing to our understanding of prebiotic chemistry in astrophysical environments.

## Supplementary Material





## Notes

Tensors in PAF, alanine labeling method, mass spectrometric analysis, X-ray orientation of d- and l-alanine, computational model comparison, NMR satellite spectrumand NMR pulse program used for data collection (PDF)
NMR data set (XLSX)

## References

[1] Chen Y., Ma W. (2020). The origin of biological homochirality along with the origin of life. PLOS Computational Biology.

[2] Blackmond D. G. (2019). The Origin
of Biological Homochirality. Cold Spring Harbor
Perspectives in Biology.

[3] Glavin D. P., Elsila J. E., McLain H. L., Aponte J. C., Parker E. T., Dworkin J. P., Hill D. H., Connolly H. C., Lauretta D. S. (2021). Extraterrestrial amino acids and
L-enantiomeric excesses
in the CM2 carbonaceous chondrites Aguas Zarcas and Murchison. Meteoritics & Planetary Science.

[4] Burton A. S., Stern J. C., Elsila J. E., Glavin D. P., Dworkin J. P. (2012). Understanding
prebiotic chemistry through the analysis of extraterrestrial amino
acids and nucleobases in meteorites. Chem. Soc.
Rev..

[5] Elsila J. E., Aponte J. C., Blackmond D. G., Burton A. S., Dworkin J. P., Glavin D. P. (2016). Meteoritic Amino
Acids: Diversity in Compositions Reflects
Parent Body Histories. ACS Central Science.

[6] Burton A. S., Berger E. L. (2018). Insights into Abiotically-Generated
Amino Acid Enantiomeric
Excesses Found in Meteorites. Life.

[7] Ulbricht T. L. V., Vester F. (1962). Attempts to induce
optical activity with polarized
β-radiation. Tetrahedron.

[8] Garcia A. D., Meinert C., Sugahara H., Jones N. C., Hoffmann S. V., Meierhenrich U. J. (2019). The astrophysical
formation of asymmetric molecules
and the emergence of a chiral bias. Life.

[9] Thiemann W. (1984). Speculations
and facts on the possible inductions of chirality through earth magnetic
field. Origins of life 1984 14:1.

[10] Quack M. (2002). How Important
is Parity Violation for Molecular and Biomolecular Chirality?. Angew. Chem., Int. Ed..

[11] Famiano M. A., Boyd R. N., Onaka T., Kajino T. (2021). Chiral selection, isotopic
abundance shifts, and autocatalysis of meteoritic amino acids. Physical Review Research.

[12] Famiano M. A., Boyd R. N., Kajino T., Onaka T. (2018). Selection of Amino
Acid Chirality via Neutrino Interactions with 14 N in Crossed Electric
and Magnetic Fields. ASTROBIOLOGY.

[13] Famiano M. A., Boyd R. N., Kajino T., Onaka T., Mo Y. (2018). Amino Acid
Chiral Selection Via Weak Interactions in Stellar Environments: Implications
for the Origin of Life. Sci. Rep..

[14] Spiess H., Garrett B., Sheline R., Rabideau S. (1969). Oxygen-17
Quadrupole
Coupling Parameters for Water in Its Various Phases. J. Chem. Phys..

[15] Hunston R. N., Gerothanassis I. P., Lauterwein J. (1985). A study of L-proline, sarcosine,
and the cis/trans isomers of N-acetyl-L-proline and N-acetylsarcosine
in aqueous and organic solution by oxygen-17 NMR. J. Am. Chem. Soc..

[16] Paquin R., Pelupessy P., Duma L., Gervais C., Bodenhausen G. (2010). Determination
of the antisymmetric part of the chemical shift anisotropy tensor
via spin relaxation in nuclear magnetic resonance. J. Chem. Phys..

[17] Anet F. A., O’Leary D. J., Wade C. G., Johnson R. D. (1990). NMR relaxation by
the antisymmetric component of the shielding tensor: a longer transverse
than longitudinal relaxation time. Chemical
physics letters.

[18] Wu G. (2008). Solid-state
17O NMR studies of organic and biological molecules. Prog. Nucl. Magn. Reson. Spectrosc..

[19] Wi S., Frydman L. (2002). quadrupolar-shielding
cross-correlations in solid state
nuclear magnetic resonance: Detecting antisymmetric components in
chemical shift tensors. J. Chem. Phys..

[20] Ashbrook S. E., Smith M. E. (2006). Solid state 17O
NMR—an introduction to the background
principles and applications to inorganic materials. Chem. Soc. Rev..

[21] Wu G. (2019). 17O NMR studies
of organic and biological molecules in aqueous solution and in the
solid state. Prog. Nucl. Magn. Reson. Spectrosc..

[22] Špačková J., Goldberga I., Yadav R., Cazals G., Lebrun A., Verdié P., Métro T., Laurencin D. (2023). Fast and Cost-Efficient ^17^ O-Isotopic Labeling of Carboxylic Groups in Biomolecules:
From Free Amino Acids to Peptide Chains. Chem.
– A Eur. J..

[23] Yamauchi K., Kuroki S., Ando I., Ozaki T., Shoji A. (1999). 17O NMR chemical
shifts and quadrupole coupling constants in solid poly (L-alanine)­s
determined using a high-speed MAS technique. Chemical physics letters.

[24] Pike K. J., Lemaitre V., Kukol A., Anupõld T., Samoson A., Howes A. P., Watts A., Smith M. E., Dupree R. (2004). Solid-State ^17^ O NMR of Amino Acids. J. Phys. Chem. B.

[25] Wu G., Dong S. (2001). Two-Dimensional 17O Multiple Quantum Magic-Angle Spinning NMR of
Organic Solids. J. Am. Chem. Soc..

[26] Wilson C. C., Myles D., Ghosh M., Johnson L. N., Wang W. (2005). Neutron diffraction
investigations of l- and d-alanine at different temperatures: the
search for structural evidence for parity violation. New J. Chem..

[27] Perdew J. P., Ruzsinszky A., Csonka G. I., Vydrov O. A., Scuseria G. E., Constantin L. A., Zhou X., Burke K. (2008). Restoring the Density-Gradient
Expansion for Exchange in Solids and Surfaces. Phys. Rev. Lett..

[28] Pickard C. J., Mauri F. (2001). All-electron magnetic
response with pseudopotentials: NMR chemical
shifts. Phys. Rev. B.

[29] Haeberlen, U. High Resolution NMR in Solids Selective Averaging. In Advances in Magnetic Resonance; Academic Press, 2012; Vol. 1, Section: xiii, 190 pages: illustrations; 24 cm.

[30] Vosegaard T. (2021). Single-crystal
NMR spectroscopy. Prog. Nucl. Magn. Reson. Spectrosc..

[31] Agarwal, S. ; Li, Z. ; Kitchen, J. ; Wi, S. ; Miller, J. B. Probing the 11B quadrupolar and chemical shielding tensors in a pair of organoboron enantiomers. J. Phys. Chem. A Accepted for publication. 10.1021/acs.jpca.5c03645. PMC12641489 41218193

[32] Vosegaard T., Skibsted J., Bildso̷e H., Jakobsen H. J. (1996). Quadrupole Coupling
and Anisotropic Shielding from Single-Crystal NMR of the Central Transition
for quadrupolar Nuclei.87Rb NMR of RbClO4and Rb2SO4. J. Magnet. Reson. Ser. A.

[33] Wu G., Zhu J. (2012). NMR studies of alkali metal ions in organic and biological solids. Prog. Nucl. Magn. Reson. Spectrosc..

[34] Millot Y., Man P. P. (2012). Active and passive
rotations with Euler angles in NMR. Concepts
Magn. Reson. A.

[35] Pouchert, C. ; Behnke, J. The Aldrich library of ^13^C and ^1^H FT NMR spectra; Aldrich Chemical Co, 1993, p. 1085.

[36] Smallman, R. ; Ngan, A. Modern Physical Metallurgy; Elsevier, 2014; pp. 159–250.

[37] Altomare A., Cuocci C., Giacovazzo C., Moliterni A., Rizzi R., Corriero N., Falcicchio A. (2013). lt EXPO2013:
a kit of tools for phasing crystal structures from powder data. J. Appl. Crystallogr..

[38] Altomare A., Campi G., Cuocci C., Eriksson L., Giacovazzo C., Moliterni A., Rizzi R., Werner P.-E. (2009). Advances
in powder
diffraction pattern indexing: lt N-TREOR09. J. Appl. Crystallogr..

[39] Altomare A., Giacovazzo C., Guagliardi A., Moliterni A. G. G., Rizzi R., Werner P.-E. (2000). New techniques for
indexing: lt N-TREOR
in lt EXPO. J. Appl. Crystallogr..

[40] Gor’kov P. L., Chekmenev E. Y., Li C., Cotten M., Buffy J. J., Traaseth N. J., Veglia G., Brey W. W. (2007). Using low-E resonators
to reduce RF heating in biological samples for static solid-state
NMR up to 900 MHz. J. Magn. Reson..

[41] Vosegaard T., Langer V., Daugaard P., Hald E., Bildsoe H., Jakobsen H. J. (1996). A new goniometer
design for single-crystal nuclear
magnetic resonance spectroscopy. Rev. Sci. Instrum..

[42] Izumi F., Momma K. (2011). VESTA 3 for three-dimensional visualization of crystal, volumetric
and morphology data. J. Appl. Crystallogr..

[43] Björkman T. (2011). CIF2Cell:
Generating geometries for electronic structure programs. Comput. Phys. Commun..

[44] Veinberg S. L., Friedl Z. W., Lindquist A. W., Kispal B., Harris K. J., O’Dell L. A., Schurko R. W. (2016). 14N Solid-State NMR Spectroscopy
of Amino Acids. ChemPhysChem.

[45] Hammer B., Hansen L. B., Norskov J. K. (1999). Improved
adsorption energetics within
density-functional theory using revised Perdew-Burke-Ernzerhof functionals. Phys. Rev. B.

[46] Monkhorst H. J., Pack J. D. (1976). Special points for
Brillouin-zone integrations. Phys. Rev. B.

[47] Profeta M., Mauri F., Pickard C. J. (2003). Accurate
First Principles Prediction
of 17O NMR Parameters in SiO2: Assignment of the Zeolite Ferrierite
Spectrum. J. Am. Chem. Soc..

[48] Yates J. R., Pickard C. J., Mauri F. (2007). Calculation
of NMR Chemical Shifts
for extended systems using Ultrasoft Pseudopotentials. Phys. Rev..

[49] Green T., Yates J. (2014). Relativistic nuclear
magnetic resonance J-coupling with ultrasoft
pseudopotentials and the zeroth-order regular approximation. J. Chem. Phys..

[50] Bonhomme C., Gervais C., Babonneau F., Coelho C., Pourpoint F., Azais T., Ashbrook S. E., Griffin J. M., Yates J. R., Mauri F., Pickard C. J. (2012). First-Principles
Calculation of NMR
Parameters Using the Gauge Including Projector Augmented Wave Method:
A Chemist’s Point of View. Chem. Rev..

[51] Clark S. J., Segall M. D., Pickard C. J., Hasnip P. J., Probert M. J., Refson K., Payne M. (2005). First principles
methods using CASTEP. Z. Kristallogr..

[52] Hohenberg P., Kohn W. (1964). Inhomogeneous electron
gas. Physical review.

[53] Kohn W., Sham L. J. (1965). Self-consistent equations including
exchange and correlation
effects. Phys. Rev..

[54] Francis G. P., Payne M. C. (1990). Finite basis set
corrections to total energy pseudopotential
calculations. J. Phys.-Condens. Matter.

[55] Tkatchenko A., Scheffler M. (2009). Accurate Molecular
Van Der Waals Interactions from
Ground-State Electron Density and Free-Atom Reference Data. Phys. Rev. Lett..

[56] Payne M. C., Teter M. P., Allan D. C., Arias T., Joannopoulos J. D. (1992). Iterative
minimization techniques for ab initio total-energy calculations -
molecular-dynamics and conjugate gradients. Rev. Mod. Phys..

[57] Pfrommer B. G., Cote M., Louie S. G., Cohen M. L. (1997). Relaxation
of crystals
with the quasi-Newton method. J. Comput. Phys..

[58] Byrd R. H., Nocedal J., Schnabel R. B. (1994). Representations
of quasi-Newton matrices
and their use in limited memory methods. Math.
Prog..

[59] Vosegaard T., Hald E., Langer V., Skov H. J., Daugaard P., Bildso̷e H., Jakobsen H. J. (1998). Improved Hardware and Software for
Single-Crystal NMR Spectroscopy. J. Magn. Reson..

[60] Giavani T., Bildso̷e H., Skibsted J., Jakobsen H. J. (2004). A solid-state
14N
magic-angle spinning NMR study of some amino acids. J. Magn. Reson..

[61] Yates J. R., Pickard C. J., Payne M. C., Dupree R., Profeta M., Mauri F. (2004). Theoretical Investigation
of Oxygen-17 NMR Shielding and Electric
Field Gradients in Glutamic Acid Polymorphs. J. Phys. Chem. A.

[62] Kongsted J., Aidas K., Mikkelsen K. V., Sauer S. P. (2008). On the accuracy
of density functional theory to predict shifts in nuclear magnetic
resonance shielding constants due to hydrogen bonding. J. Chem. Theory Comput..

[63] Harris, F. E. Mathematics for Physical Science and Engineering; Elsevier, 2014; pp. 293–323.

[64] Jakobsen, H. J. ; Bildso̷e, H. ; Skibsted, J. ; Giavani, T. 14N MAS NMR Spectroscopy. An Instrumental Challenge and Informatory Technique. In Magnetic Resonance in Colloid and Interface Science, 2002; pp. 43–55.

