# Supplementary material for: Single-Crystal NMR for 17O in Alanine Enantiomers
Source: ACS Phys Chem Au. 2025 Nov 12;6(1):13–28. doi: 10.1021/acsphyschemau.5c00061 (PMC12856672; doi:10.1021/acsphyschemau.5c00061)
Supplement: Supplementary file 1 [file pg5c00061_si_001.pdf]

Supplementary Material for:

Single-Crystal NMR for ^{17}O in Alanine Enantiomers

Shiva Agarwal, Sungsool Wi, Jason Kitchen, Zhongrui Li, Christopher J. Taylor, Michael A. Famiano, and John B. Miller*

*Corresponding author: john.b.miller@wmich.edu

S-1 CS and EFG Tensor in PAF

Total CS tensor (δ_{CS}) is given as the sum of Symmetric CS ($\delta_{\mathrm{CS}}^{(s)}$) and anti-symmetric CS ($\delta_{\mathrm{CS}}^{(a)}$) tensor

$$\delta_{\mathrm{CS}} = \delta_{\mathrm{CS}}^{(s)} + \delta_{\mathrm{CS}}^{(a)} \tag{1}$$

In PAF of CS tensor,

$$\boldsymbol{\delta}_{\mathrm{PAF}}^{(s)} = \begin{pmatrix} \delta_{iso} - \frac{1}{2}(1+\eta_{CS})\delta_{CS} & 0 & 0 \\ 0 & \delta_{iso} - \frac{1}{2}(1-\eta_{CS})\delta_{CS} & 0 \\ 0 & 0 & \delta_{iso} + \delta_{CS} \end{pmatrix} \tag{2}$$

$$\boldsymbol{\delta}_{\mathrm{PAF}}^{(a)} = \begin{pmatrix} 0 & \delta_{XY}^{(a)} & \delta_{XZ}^{(a)} \\ -\delta_{XY}^{(a)} & 0 & \delta_{YZ}^{(a)} \\ -\delta_{XZ}^{(a)} & -\delta_{YZ}^{(a)} & 0 \end{pmatrix} \tag{3}$$

In its PAF, the quadrupolar coupling tensor ($\boldsymbol{Q}_{\mathrm{PAS}}$) that is related to the EFG tensor ($\boldsymbol{Q} = \frac{eQ}{\hbar}\boldsymbol{V}$) is given as

$$\mathbf{Q}_{PAS} = C_Q \begin{pmatrix} -\frac{1}{2}(1+\eta_Q) & 0 & 0 \\ 0 & -\frac{1}{2}(1-\eta_Q) & 0 \\ 0 & 0 & 1 \end{pmatrix} \tag{4}$$

S-2 ^{17}O Isotopic Labeling of Alanine

To a stirred suspension of 3.5 g (2.5 equivalents) sodium ethoxide in 10 mL methanol at 0°C was added sequentially 1.0 g (2.5 equivalents) $H_2{}^{17}O$ (CIL, 40 atom-% nominal, 41.0 atom-% d,,id,[18]Od,,id,[18]O^{17}O and 6.2 atom-% ^{18}O by analysis) then 2.8 g (1.0 equivalent) *D*-alanine methyl ester hydrochloride. The suspension was refluxed with stirring for two hours then cooled to ambient (*ca.* 22°C). Sufficient water (*ca.* 10 mL) was added to the mixture to dissolve the solids. The mixture was titrated to pH 5.5 with 10 *M* H_2SO_4(*aq.*), then adjusted to pH 6 with 1.25 *M* NaOH(*aq.*). Excess methanol (*ca.* 25 mL) was added, whereupon minor precipitation of a white solid was observed.

After standing overnight at −18°C, the white crystals were vacuum filtered with a methanol wash, then oven-dried at 60°C to yield 1.35 g of *D*-alanine-$^{17}O_1$ (*ca.* 20 atom-%). A subsequent 0.27 g crop of crystals was harvested from the supernatant after removing the solvent under vacuum, dissolving the solid in a minimum volume of water (*ca.* 5 mL), adding excess methanol (25 mL) and repeating the crystallization and harvesting procedure, giving a combined recovered yield of 89% of the labeled product.

The ^{17}O-labeled *L*-alanine was prepared similarly with equivalent yield and label incorporation.

S-3 Mass Spectrometry Spectra

Mass spectra of both samples were acquired using a linear ion trap (LTQ XL) mass spectrometer equipped with an IonMax electrospray ionization (ESI) source (Thermo Scientific, Waltham, MA, USA). Aliquots (5 μ)L of 10 μM amino acid solutions, prepared in 50% methanol with 0.1% formic acid, were sprayed via loop injection at a rate of 5 μL/min. Electrospray ionization was performed at a spray voltage of 4 kV, with N_2 used as the sheath gas delivered at 10 arbitrary units. The MS inlet temperature was set to 250°C. The LTQ ion optics were set to 20 V for the ion transfer capillary and 65 V for the tube lens.

To confirm successful ^{17}O-labeling of *L*- and *D*-alanine, mass spectra were analyzed for characteristic mass shifts. The resulting spectra (see Figures S1 and S2) shows the incorporation of ^{17}O isotopes in both samples. Note that it was not the intent of this measurement to precisely quantify the extent of labeling, but generally confirm its extent of the labeling. More important was that sufficient label be added to the compound for NMR analysis.

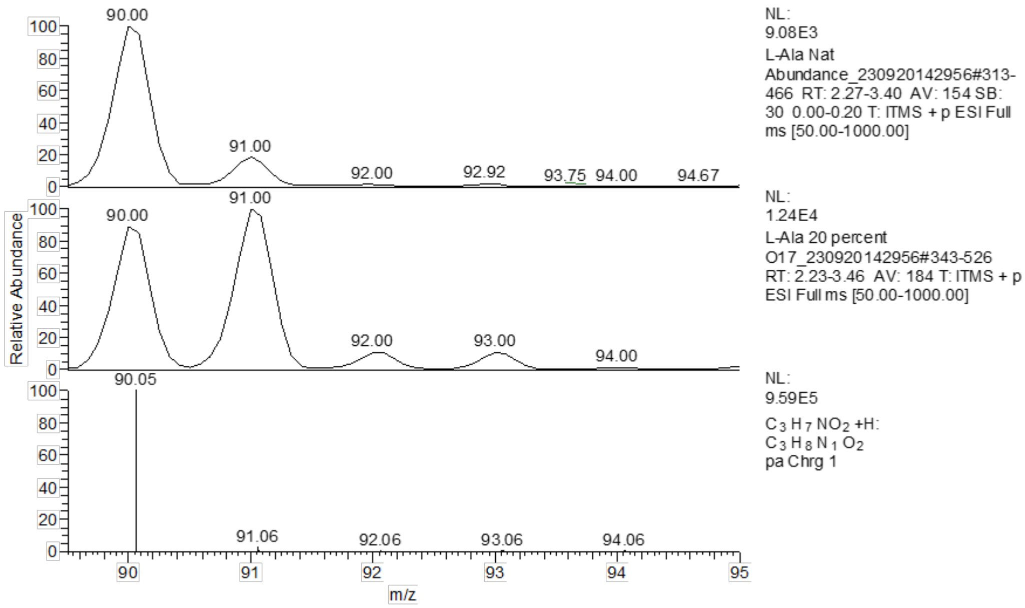

Figure S1: Spectrum showing mass spectroscopy analysis to confirm ^{17}O labeling for *L*-alanine.

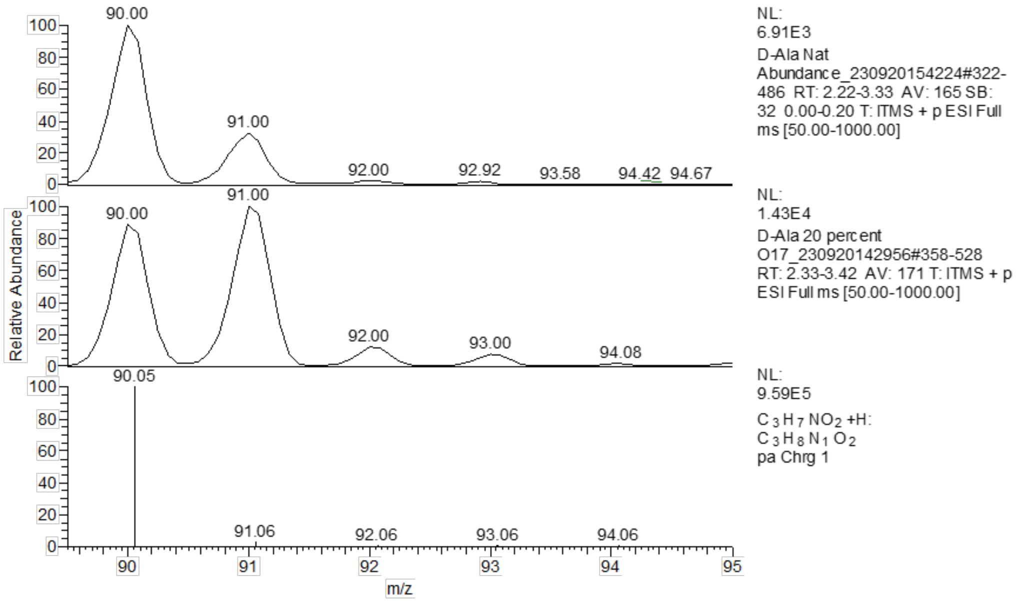

Figure S2: Spectrum showing mass spectroscopy analysis to confirm ^{17}O labeling for *D*-alanine.

S-4 Calculation of Orientation Angles

From x-ray diffraction (XRD), the orientation of a single crystal with respect to the tenon on which it was mounted, were obtained. The XRD procedure marked the surface normal direction and an edge plane of the crystal. This information was used to find the other two orthogonal axes of the tenon in terms of Miller indices (*hkl*) of the unit cell in the sample. The direction cosines of the crystal axes ($a,\ b,\ c$) with respect to the tenon axes ($X,\ Y,\ Z$) were then specified. The determination of Euler angles (α, β, γ) for orthorhombic crystal structure of alanine was performed using TRAFO [1].

S-4.1 Orientation of *L*-alanine

From XRD the surface normal of *L*-alanine was determined to be in the direction (0 2 3) and the edge plane was oriented toward (1 0 0) (See Figure S3).

(a)

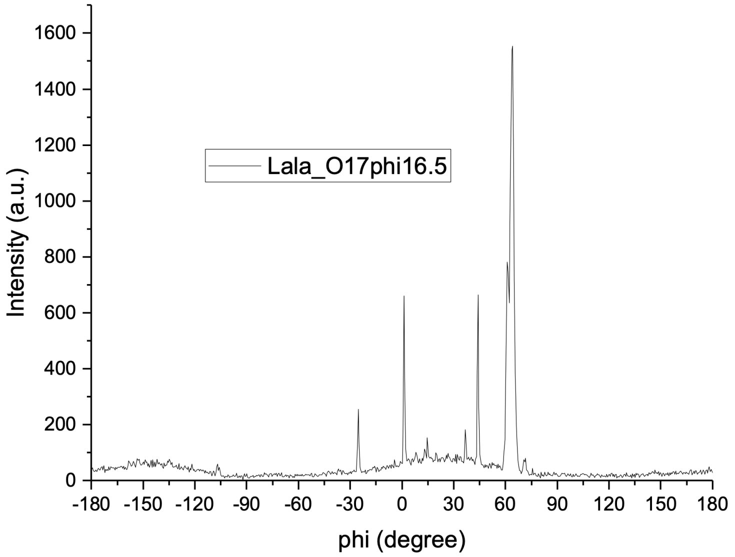

(b)

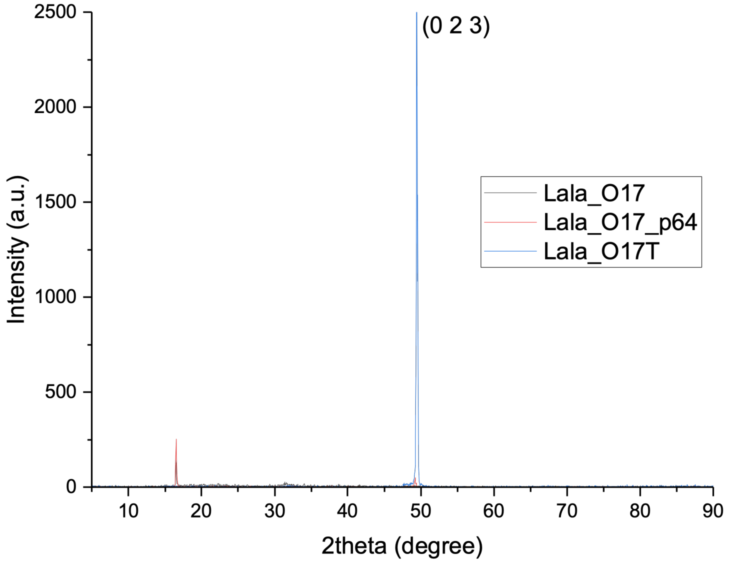

Figure S3: Experimental X-ray diffraction patterns showing **(a)** Intensity vs. ϕ and **(b)** Intensity vs. 2θ plots for L-alanine single crystal.

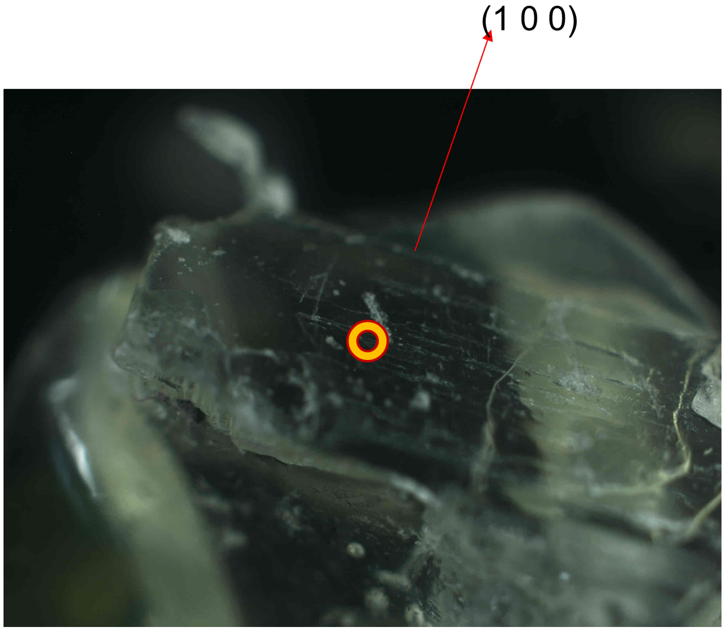

Figure S4: The circle in the picture marks the surface normal direction (0 2 3) and the red arrow shows the edge plane (1 0 0) of the *L*-alanine single crystal.

A fiduciary dot on the tenon was marked so that the X direction of the plate could be fixed. The Y axis of the plate is perpendicular to this direction. The angle between (1 0 0) and tenon X direction was found to be $63.0° \pm 0.5°$ (See Figure S5)

Find X direction as crystallographic vector

Let $(x_1\ x_2\ x_3)$ be a unit vector making an angle of 63° with (1 0 0) vector. The dot product of both vectors gives:

$$(1\ 0\ 0) \cdot (x_1\ x_2\ x_3) = \cos 63°$$
$$x_1 \approx 0.45$$

The surface normal or **Z** vector is perpendicular to **X** vector

$$\implies \mathbf{Z} \cdot \mathbf{Y} = 0$$
$$(0\ 2\ 3) \cdot (x_1\ x_2\ x_3) = 0$$
$$2x_2 = -3x_3$$

Since **X** must be a unit vector

$$
\begin{aligned}
x_1^2 + x_2^2 + x_3^2 &= 1 \\
(0.45)^2 + (-1.5x_3)^2 + x_3^2 &= 1 \\
x_3 &\approx \pm 0.50 \\
\text{taking } x_3 &= 0.50 \\
x_2 &= -0.75
\end{aligned}
$$

$$\mathbf{X} = (0.45\ \ -0.75\ 0.50)$$

In terms of crystallographic directions,

$$\mathbf{X} = (9\ \ -15\ 10)$$

The angle θ between (1 0 0) and **X** can be verified as

$$
\begin{aligned}
(1\ 0\ 0) \cdot \mathbf{X} &= |(1\ 0\ 0)||\mathbf{X}| \cos\theta \\
\theta &= \cos^{-1}(9/\sqrt{406}) \\
&\approx 63.5^\circ
\end{aligned}
$$

Find Y direction as crystallographic vector

Since $\mathbf{X}, \mathbf{Y}, \mathbf{Z}$ vectors are orthogonal to each other

$$
\begin{aligned}
\mathbf{Y} &= \mathbf{Z} \times \mathbf{X} \\
&= (0\ 2\ 3) \times (9\ 15\ 10) \\
&= (65\ 27\ \ -18)
\end{aligned}
$$

The angle ϕ between (1 0 0) and **Y** can be verified as

$$
\begin{aligned}
(1\ 0\ 0) \cdot \mathbf{Y} &= |(1\ 0\ 0)||\mathbf{Y}| \cos\phi \\
\phi &= \cos^{-1}(1/\sqrt{5278}) \\
&= 26.5^\circ
\end{aligned}
$$

The crystal axes and the tenon directions on the unit cell of$L$-alanine were visualized in *VESTA* [3] (see Figure S6) to verify that they represent the measured crystal axes and the physical plate directions.

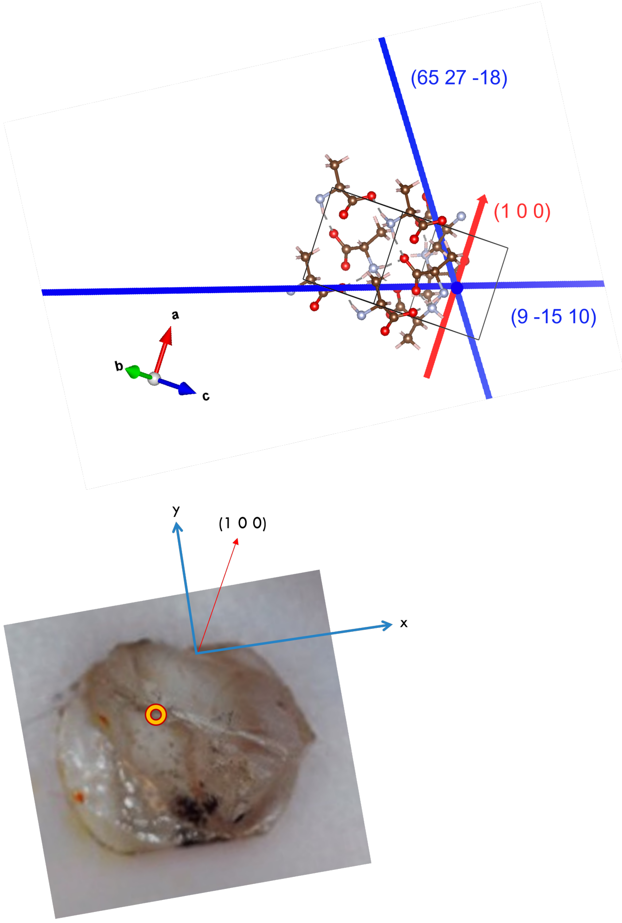

Figure S6: Crystal axes represented in red and tenon axes represented in blue color. *Left:* Representation of axes in the unit cell of *L*-alanine. *Right:* Representation of axes on the single crystal of *L*-alanine.

The direction cosines of the crystal axes $(a,\ b,\ c)$ with respect to the tenon axes $(X,\ Y,\ Z)$ were calculated to be

	X axis	Y axis	Z axis
a axis	$\frac{9}{\sqrt{406}}$	$\frac{65}{\sqrt{5278}}$	$\frac{0}{\sqrt{13}}$
b axis	$\frac{-15}{\sqrt{406}}$	$\frac{27}{\sqrt{5278}}$	$\frac{2}{\sqrt{13}}$
c axis	$\frac{10}{\sqrt{406}}$	$\frac{-18}{\sqrt{5278}}$	$\frac{3}{\sqrt{13}}$

Using TRAFO, the triple of Euler angles that specify the orientation of

the orthogonalized crystal axis system in the tenon frame was $(333.5°, 33.7°, 90.0°)$.

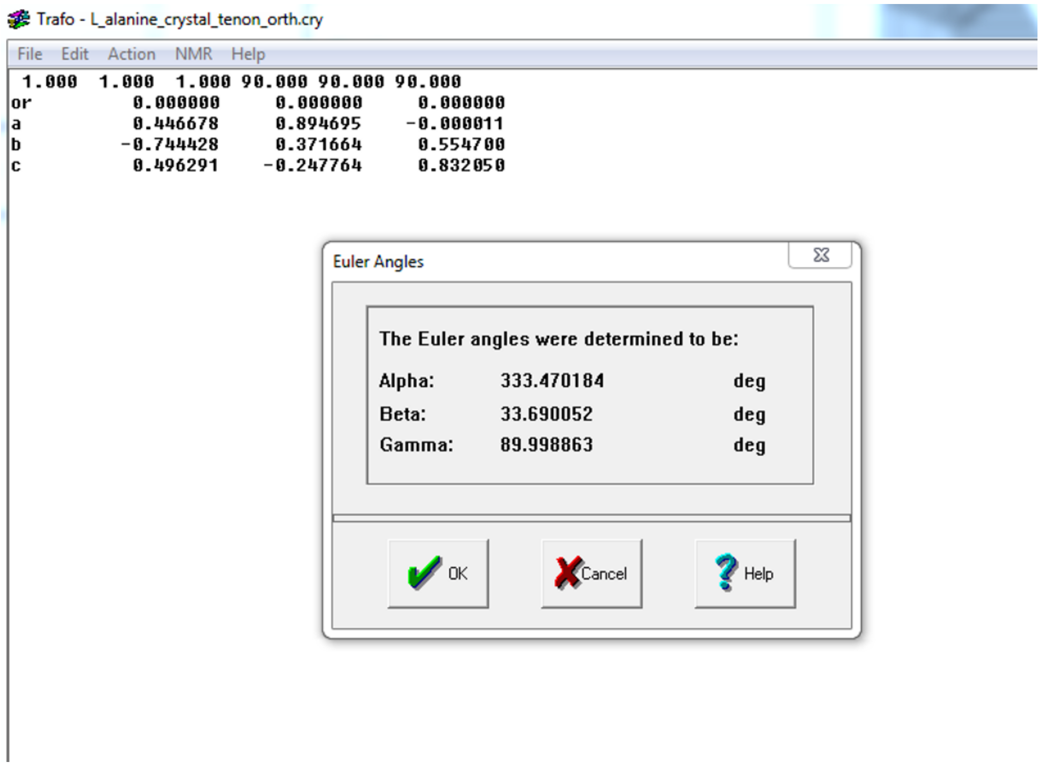

Figure S7: Calculation of orientation angles for *L*-alanine in TRAFO

For transformation from frame A with basis $\{X_A, Y_A, Z_A\}$ to frame B with basis $\{X_B, Y_B, Z_B\}$, passive rotation by an angle α around the Z_A axis is performed to result in an intermediate frame $\{X'_A, Y'_A, Z_A\}$. The first rotation is followed by a passive rotation by an angle β around the new Y'_A axis that results in frame $\{X''_A, Y'_A, Z_B\}$. Finally, passive rotation around Z_B by an angle γ is carried out. Figure S8 shows the step-wise rotations as described. All rotations are counter clockwise.

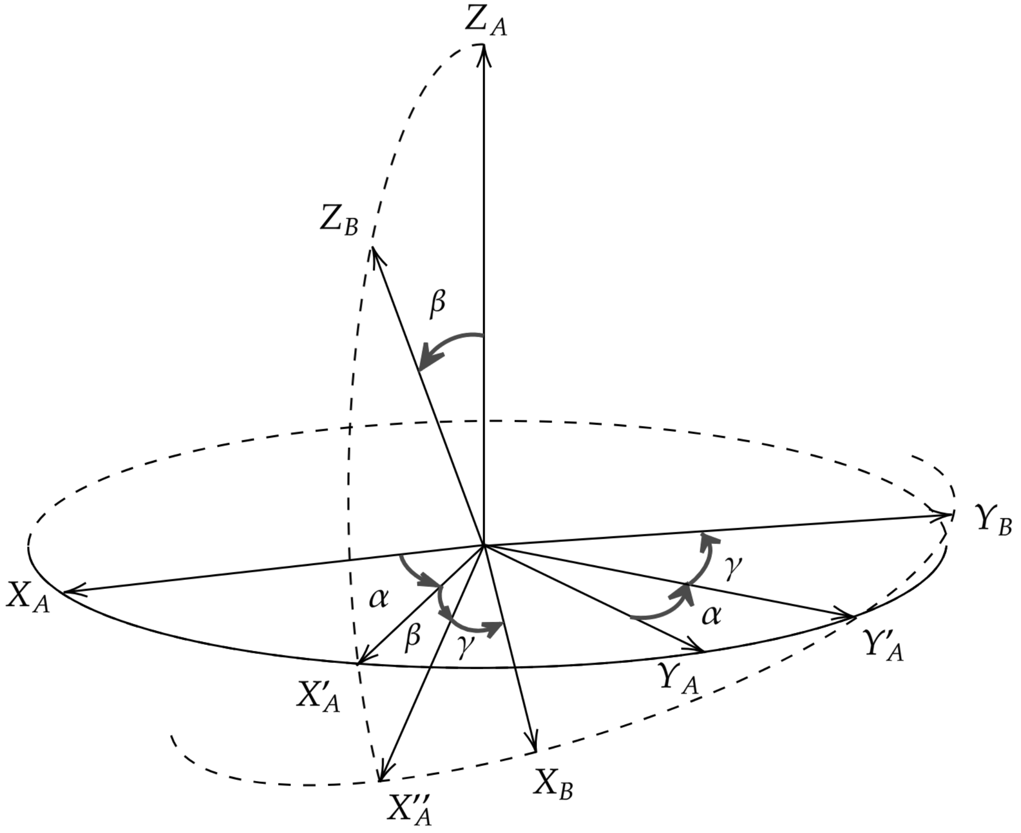

Figure S8: Transformation of frame A to frame B using rotations defined by the Euler triplet (α, β, γ).

S-5 Orientation of *D*-alanine

From XRD the surface normal of *D*-alanine was determined to be in the direction (1 1 0) and the edge plane (C-axis) was oriented toward (0 0 1).

(a)

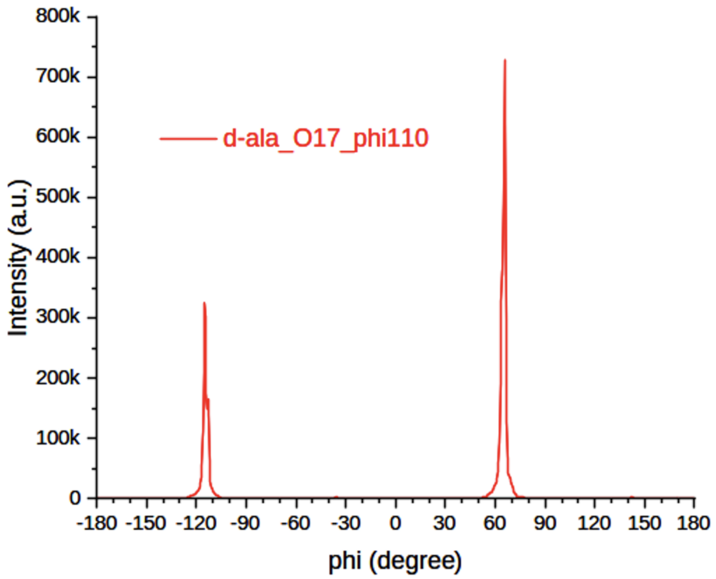

(b)

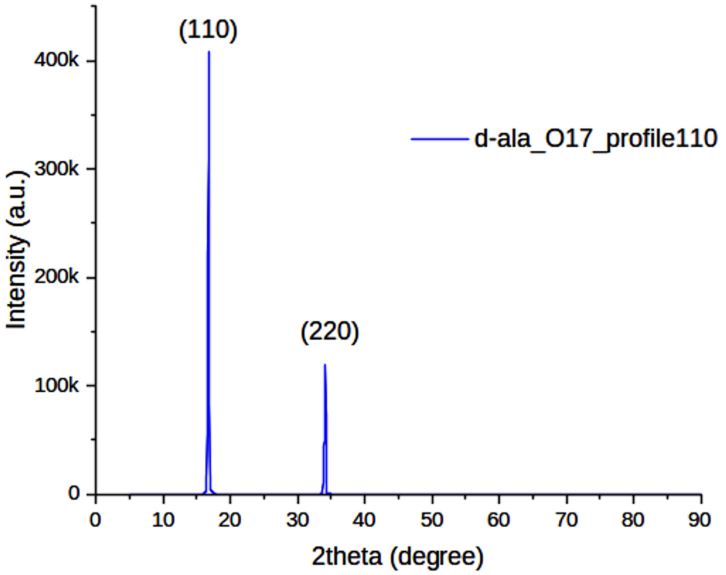

Figure S9: Experimental X-ray diffraction patterns showing **(a)** Intensity vs. ϕ and **(b)** Intensity vs. 2θ plots for the D-alanine single crystal.

The angle between (0 0 1) and tenon X direction was found to be 6.0°±0.5°

Find X direction as crystallographic vector

Let $(x_1\ x_2\ x_3)$ be a unit vector making an angle of 6° with (0 0 1) vector. The dot product of both vectors gives:

$$\begin{aligned}(0\ 0\ 1) \cdot (x_1\ x_2\ x_3) &= \cos 6^\circ \\ x_1 &\approx 0.99\end{aligned}$$

The surface normal or **Z** vector is perpendicular to **X** vector

$$\begin{aligned}\implies \mathbf{Z} \cdot \mathbf{Y} &= 0 \\ (1\ 1\ 0) \cdot (x_1\ x_2\ x_3) &= 0 \\ x_2 &= -x_1\end{aligned}$$

Since **X** must be a unit vector

$$\begin{aligned}x_1^2 + x_2^2 + x_3^2 &= 1 \\ (x_1)^2 + (-x_1)^2 + 0.99^2 &= 1 \\ x_1 &\approx \pm 0.09 \\ \text{taking } x_1 &= 0.09 \\ x_2 &= -0.09 \\ \mathbf{X} = (0.09\ &-0.09\ 0.99)\end{aligned}$$

In terms of crystallographic directions,

$$\mathbf{Y} = (1\ \ -1\ 11)$$

The angle θ between (0 0 1) and **X** can be verified as

$$\begin{aligned}(0\ 0\ 1) \cdot \mathbf{X} &= |(0\ 0\ 1)||\mathbf{X}| \cos \theta \\ \theta &= \cos^{-1}(11/\sqrt{123}) \\ &\approx 7.3^\circ\end{aligned}$$

Find Y direction as crystallographic vector

Since $\mathbf{X}, \mathbf{Y}, \mathbf{Z}$ vectors are orthogonal to each other

$$\begin{aligned}\mathbf{Y} &= \mathbf{Z} \times \mathbf{X} \\ &= (1\ 1\ 0) \times (1\ \ -1\ 11) \\ &= (11\ \ -11\ \ -2)\end{aligned}$$

The angle ϕ between $(0\ 0\ 1)$ and $\mathbf{Y}$ can be verified as

$$\begin{aligned}(0\ 0\ 1) \cdot \mathbf{Y} &= |(0\ 0\ 0)||\mathbf{Y}|\ \cos\phi \\ \phi &= \cos^{-1}(-2/\sqrt{246}) \\ &= 97.3^{\circ}(\text{or} - 82.7^{\circ})\end{aligned}$$

The calculated crystal and tenon axes agreed well with the measured axes (Figure S12).

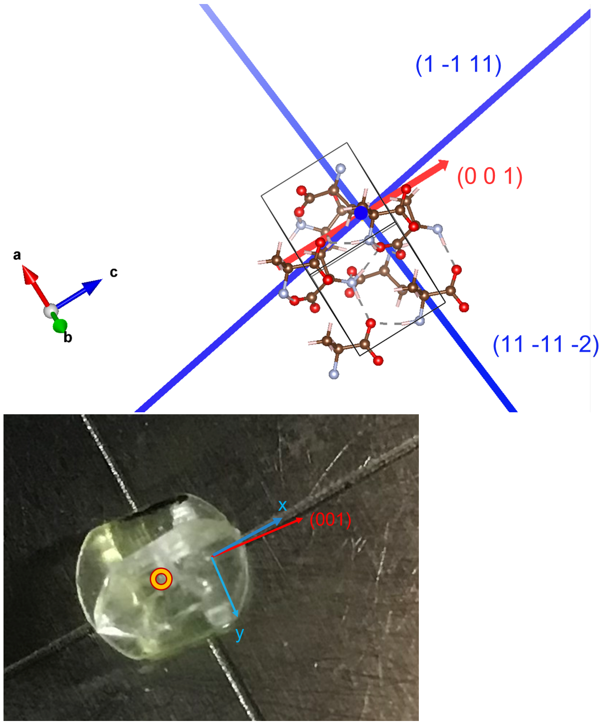

Figure S12: Crystal axes represented in red and tenon axes represented in blue color. *Left:* Representation of axes in the unit cell of *D*-alanine. *Right:* Representation of axes on the single crystal of *D*-alanine.

The direction cosines of the crystal axes (a, b, c) with respect to the tenon axes (X, Y, Z) were calculated to be

	X axis	Y axis	Z axis
a axis	$\frac{1}{\sqrt{123}}$	$\frac{11}{\sqrt{246}}$	$\frac{1}{\sqrt{2}}$
b axis	$\frac{-1}{\sqrt{123}}$	$\frac{-11}{\sqrt{246}}$	$\frac{1}{\sqrt{2}}$
c axis	$\frac{11}{\sqrt{123}}$	$\frac{-2}{\sqrt{246}}$	$\frac{0}{\sqrt{2}}$

Using TRAFO, the triple of Euler angles that specify the orientation of the orthogonalized crystal axis system in the tenon frame was $(352.7°, 90.0°, 135.0°)$.

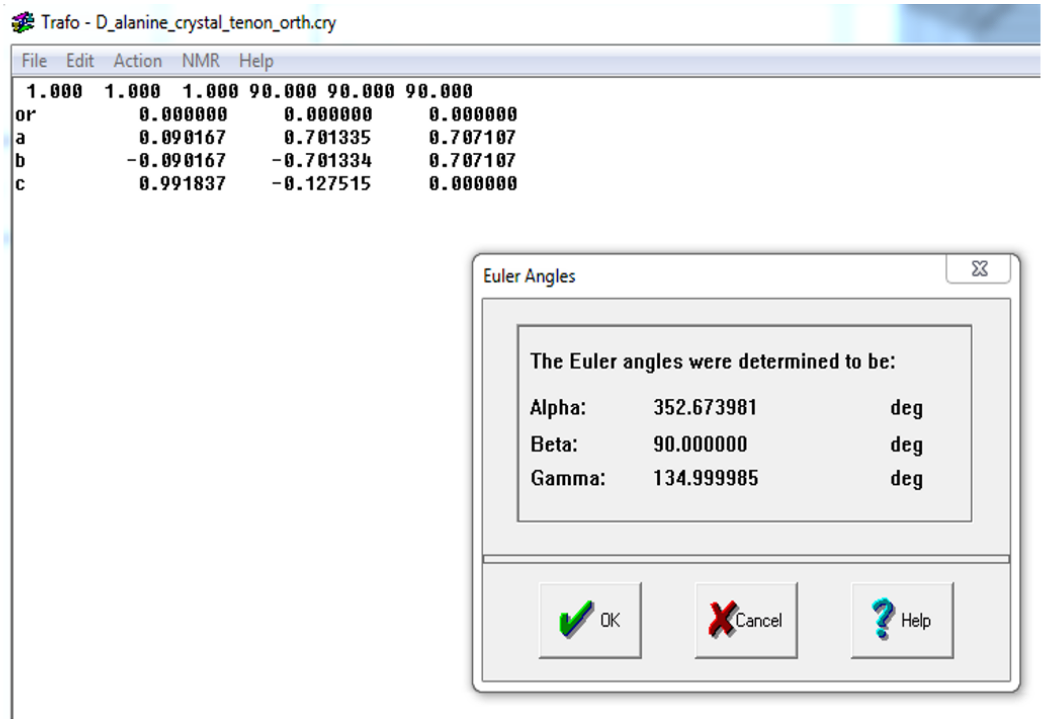

Figure S13: Calculation of orientation angles for D-alanine in TRAFO

S-6 Computational Model Comparison

The NMR parameters were calculated for optimized (see Table 1) and unoptimized (see Table 2) structures of literature neutron-diffraction crystal structures of *L*-alanine and *D*-alanine. The calculations were performed using PBEsol and rPBE functional. The Electric Field Gradient (EFG) and nuclear magnetic shielding tensors were then calculated using ultra-soft pseudopotentials using the GIPAW approach.

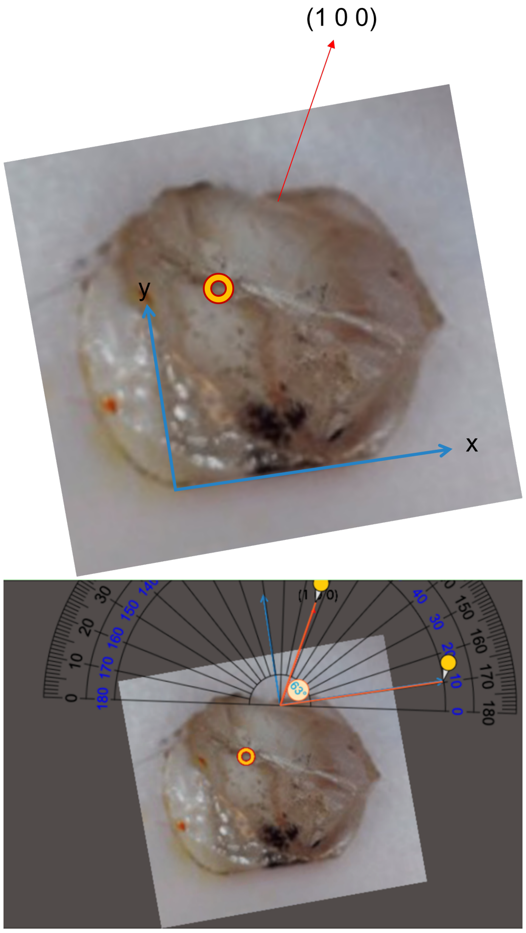

Figure S5: *Left:* Top view of the crystal showing tenon with the glued crystal. A mark representing X direction of the plate can be seen at the bottom of the plate. The mark was used as a guide to set the X and Y direction of the plate. *Right:* Measurement of angle between (1 0 0) and X vector using online protractor tool [2].

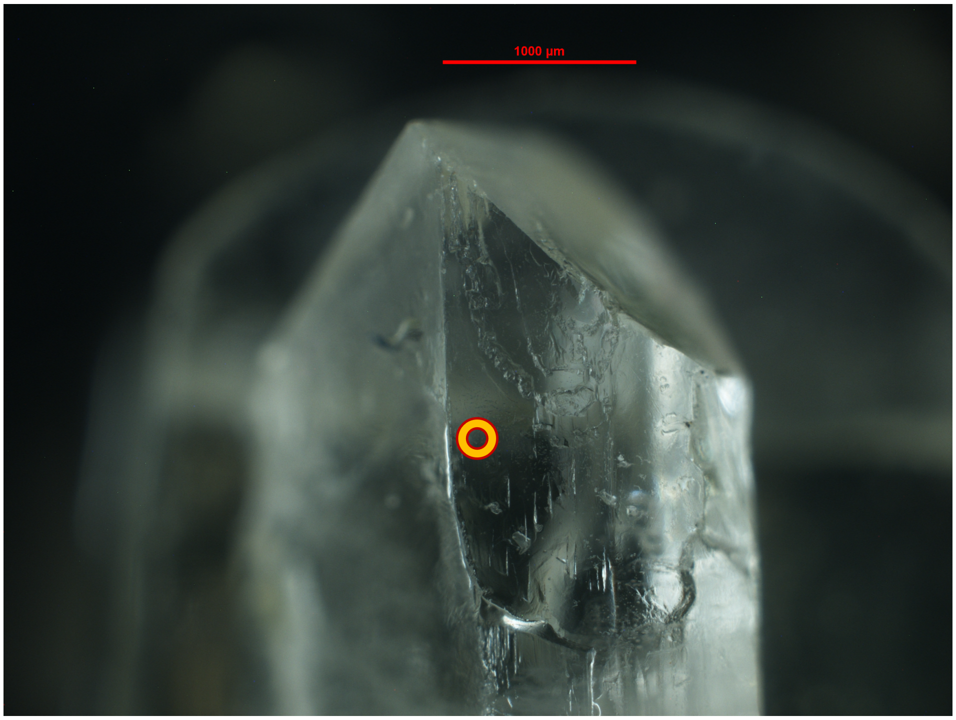

Figure S10: The circle in the picture marks the surface normal direction (1 1 0) of *D*-alanine single crystal.

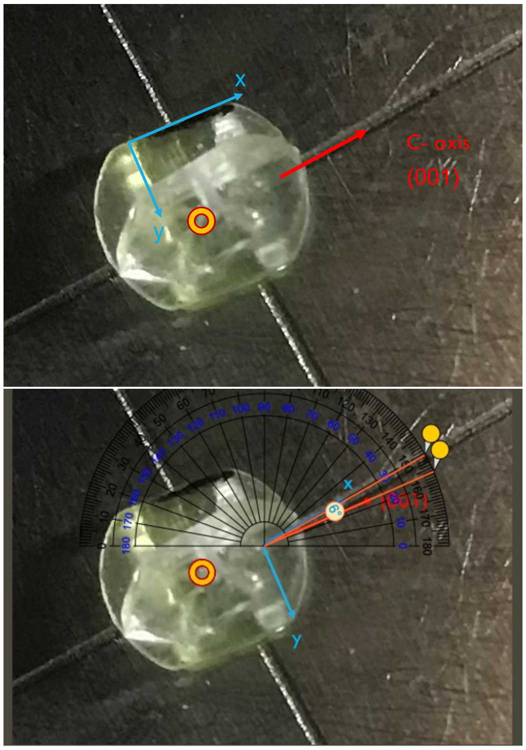

Figure S11: *Left:* Top view of the crystal showing tenon with the glued crystal. A mark representing X direction of the plate can be seen at the top of the plate. The mark was used as a guide to set the X and Y direction of the plate. The red arrow shows the edge plane (C-axis). *Right:* Measurement of angle between (0 0 1) and X vector using online protractor tool.

Structure	Nucleus	Site	$\lvert C_Q \rvert$ / MHz	η_Q	δ_{CS} / ppm	η_{CS}	$\delta_{iso}{}^{\dagger}$ / ppm	a /deg	b /deg	c /deg
**PBEsol + GIPAW**										
278464	^{17}O	1	8.34	0.25	298	0.47	-41.8	138	89	98
		2	6.36	0.77	198	0.72	-20.8	29	87	93
	^{14}N	-	1.39	0.39	10	0.96	184.2	51	41	138
**rPBE + GIPAW**										
278464	^{17}O	1	8.60	0.28	294	0.45	-34.1	139	89	97
		2	6.53	0.81	198	0.71	-12.1	28	87	92
	^{14}N	-	1.32	0.43	9	0.87	185.5	126	140	138

† Unreferenced

Table 1: Comparison of computed NMR parameters of ^{17}O and ^{14}N in optimized structures for *L*-alanine at 295 K (278464.cif).

Structure	Nucleus	Site	$\lvert C_Q \rvert$ / MHz	η_Q	δ_{CS} / ppm	η_{CS}	$\delta_{iso}{}^{\dagger}$ / ppm	a /deg	b /deg	c /deg
**PBEsol + GIPAW**										
278464	^{17}O	1	8.09	0.22	282	0.48	-30.7	39	89	97
		2	6.49	0.65	202	0.68	-11.9	29	88	95
	^{14}N	-	1.29	0.25	10	0.99	186.4	125	150	150
278466	^{17}O	1	8.17	0.26	284	0.50	-37.3	40	91	97
		2	6.6	0.65	209	0.66	-18.2	150	88	86
	^{14}N	-	1.36	0.23	11	0.84	186.9	60	148	36
278467	^{17}O	1	8.19	0.26	286	0.49	-40.3	140	89	97
		2	6.59	0.69	207	0.68	-20.3	29	88	94
	^{14}N	-	1.33	0.22	11	0.95	185.3	121	148	144
**rPBE + GIPAW**										
278464	^{17}O	1	8.26	0.26	276	0.46	-18.1	142	89	96
		2	6.63	0.69	200	0.66	0.6	27	88	94
	^{14}N	-	1.21	0.29	10	0.91	188.0	123	151	150
278466	^{17}O	1	8.36	0.30	278	0.48	-24.4	38	91	96
		2	6.74	0.69	207	0.65	-5.4	151	88	86
	^{14}N	-	1.28	0.26	11	0.77	188.7	63	149	36
278467	^{17}O	1	8.37	0.29	280	0.47	-27.3	141	89	96
		2	6.73	0.72	205	0.67	-7.5	28	88	94
	^{14}N	-	1.25	0.25	10	0.89	187.0	119	149	143

† Unreferenced

Table 2: Comparison of computed NMR parameters of ^{17}O and ^{14}N in unoptimized structures for *L*-alanine at 295 K (278464.cif), 60 K (278467.cif), and *D*-alanine at 60 K (278466.cif).

S-7 Satellite Transitions

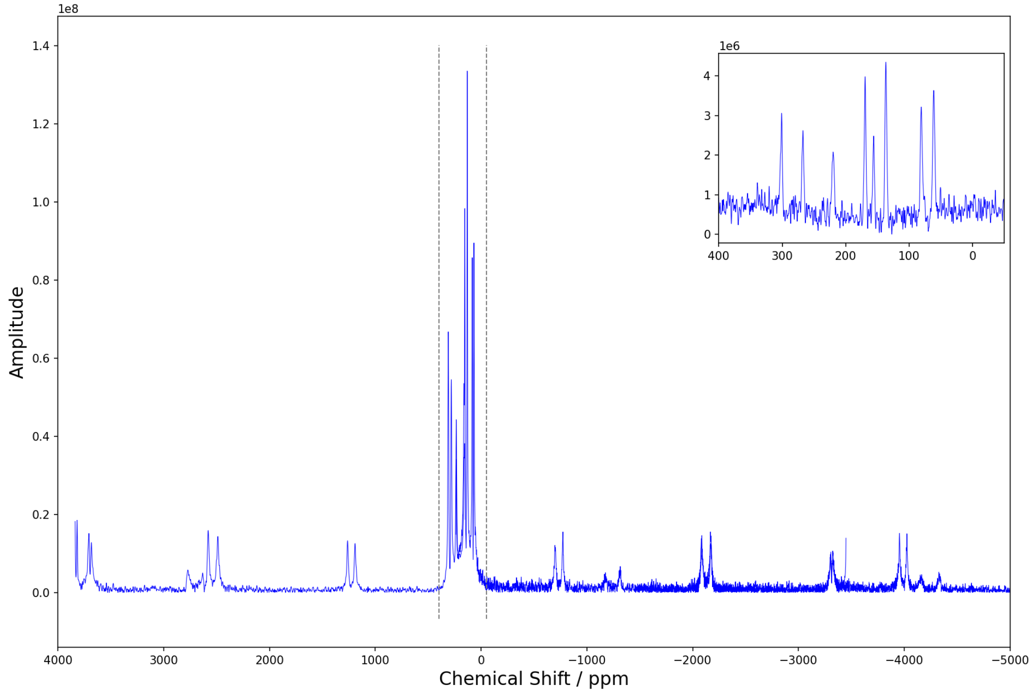

Figure S14: ^{17}O NMR spectrum acquired for L-alanine sample about -x^T at 30° rotation angle. The spectrum is created by combining three spectra at 2000 ppm, -1600 ppm, and -3300 ppm frequency offset respectively. The inset shows magnified region between the gray colored lines for the central transitions collected at 0 ppm frequency offset.

S-8 Code Listings

```
;zg
;avance-version (12/01/11)
;1D sequence
;
;$CLASS=HighRes
;$DIM=1D
;$TYPE=
;$SUBTYPE=
;$COMMENT=


#include <Avance.incl>


"acqt0=-p1*2/3.1416"


1 ze
2 30m do:f2
  d1
  (5u pl1):f1
  5u pl22:f2
  p1 ph1
  0.5u cpds1:f2
  go=2 ph31
  1m do:f2
  30m mc #0 to 2 F0(zd)
exit


ph1=0 2 2 0 1 3 3 1
ph31=0 2 2 0 1 3 3 1
```

Listing S1: dpdec.swi pulse program used for data collection

References

[1] Klaus Eichele. TRAFO — Coordinate Transformations, 2021.

[2] Ginifab online Protractor.

[3] F. Izumi and K. Momma. VESTA 3 for three-dimensional visualization of crystal, volumetric and morphology data. *Journal of Applied Crystallography*, 44(6):1272–1276, December 2011. Publisher: International Union of Crystallography.
